# Lipocalin 2 modulates dendritic cell activity and shapes immunity to influenza in a microbiome dependent manner

**DOI:** 10.1371/journal.ppat.1009487

**Published:** 2021-04-27

**Authors:** Martin L. Watzenboeck, Barbara Drobits, Sophie Zahalka, Anna-Dorothea Gorki, Asma Farhat, Federica Quattrone, Anastasiya Hladik, Karin Lakovits, Gabriel M. Richard, Therese Lederer, Birgit Strobl, Gijs A. Versteeg, Louis Boon, Philipp Starkl, Sylvia Knapp

**Affiliations:** 1 Research Laboratory of Infection Biology, Department of Medicine I, Medical University of Vienna, Austria; 2 CeMM, Research Center for Molecular Medicine of the Austrian Academy of Sciences, Austria; 3 Institute of Animal Breeding and Genetics, Department of Biomedical Science, University of Veterinary Medicine Vienna, Vienna, Austria; 4 Department of Microbiology, Immunobiology, and Genetics, Max Perutz Labs, University of Vienna, Vienna Biocenter (VBC), Vienna, Austria; 5 Polpharma Biologics, Utrecht, The Netherlands; Emory University, UNITED STATES

## Abstract

Lipocalin 2 (LCN2) is a secreted glycoprotein with roles in multiple biological processes. It contributes to host defense by interference with bacterial iron uptake and exerts immunomodulatory functions in various diseases. Here, we aimed to characterize the function of LCN2 in lung macrophages and dendritic cells (DCs) using *Lcn2*^*-/-*^ mice. Transcriptome analysis revealed strong LCN2-related effects in CD103^+^ DCs during homeostasis, with differential regulation of antigen processing and presentation and antiviral immunity pathways. We next validated the relevance of LCN2 in a mouse model of influenza infection, wherein LCN2 protected from excessive weight loss and improved survival. LCN2-deficiency was associated with enlarged mediastinal lymph nodes and increased lung T cell numbers, indicating a dysregulated immune response to influenza infection. Depletion of CD8^+^ T cells equalized weight loss between WT and *Lcn2*^-/-^ mice, proving that LCN2 protects from excessive disease morbidity by dampening CD8^+^ T cell responses. *In vivo* T cell chimerism and *in vitro* T cell proliferation assays indicated that improved antigen processing by CD103^+^ DCs, rather than T cell intrinsic effects of LCN2, contribute to the exacerbated T cell response. Considering the antibacterial potential of LCN2 and that commensal microbes can modulate antiviral immune responses, we speculated that LCN2 might cause the observed influenza phenotype via the microbiome. Comparing the lung and gut microbiome of WT and *Lcn2*^*-/-*^ mice by 16S rRNA gene sequencing, we observed profound effects of LCN2 on gut microbial composition. Interestingly, antibiotic treatment or co-housing of WT and *Lcn2*^*-/-*^ mice prior to influenza infection equalized lung CD8^+^ T cell counts, suggesting that the LCN2-related effects are mediated by the microbiome. In summary, our results highlight a novel regulatory function of LCN2 in the modulation of antiviral immunity.

## Author summary

Outcome from infectious diseases is determined by the adequacy of the immune response, with efficient clearance of the causative pathogen, while keeping the inflammatory response in check to avoid excessive inflammation, tissue damage and resulting mortality. The regulation of immunity is shaped by multiple players. As such lipocalin (LCN) 2 exhibits immune-modulatory properties and for instance deactivates alveolar macrophages, but the precise mode of action remains unclear. Here, we explored the precise effects of LCN2 on cellular lung immunity, and discovered that LCN2 markedly impacted homeostatic expression of pulmonary dendritic cell (DC) genes related to anti-influenza immunity. In agreement with this finding, LCN2-deficient mice infected with influenza virus showed a higher mortality and more pronounced immune response, despite an unaltered viral clearance. Mechanistically, we found the presence of LCN2 associated with dampened DC functions, ultimately preventing exuberant T cell activation during influenza infection. Considering LCN2’s antibacterial effects as a siderophore-binding protein, we speculated that an indirect, microbiome-dependent effect might explain these findings. In fact, LCN2 remarkably shaped the microbiome composition, and interference via antibiotics or cohousing erased the protective effects of LCN2 on the anti-viral immune response.

## Introduction

Lipocalin 2 (LCN2) is a 25-kDa secreted glycoprotein involved in a variety of biological processes, including immune responses, iron homeostasis and metabolism [1–3]. Acting as scavenger of bacterial siderophores, LCN2 is known as host defense molecule with potent anti-bacterial activity against siderophore-dependent bacteria, such as *Escherichia coli* and *Klebsiella pneumonia* [4,5]. In line with this function, LCN2 is highly expressed in barrier tissues exposed to microorganisms including the lungs [6] and further induced upon Toll-like receptor (TLR) stimulation [4].

Multiple studies have uncovered immunoregulatory effects of LCN2 beyond scavenging bacterial siderophores, and LCN2 has been found to protect from excessive inflammation-related morbidity in sterile endotoxemia [7], neuroinflammation [8] and non-alcoholic steatohepatitis (NASH) [9]. Intriguingly, all these observations are linked to effects of LCN2 on myeloid cells, such as macrophages or, in the context of NASH, macrophage-neutrophil interplay. Our group could previously show that LCN2 deactivates lung macrophages and worsens disease outcome from pneumonia caused by the siderophore-independent pathogen *Streptococcus pneumoniae* [10]. These studies highlight effects of LCN2 on myeloid cell function and plasticity, with important consequences in various infectious and non-infectious diseases. Given the expression pattern at mucosal surfaces including the intestine and LCN2’s potential to alter the availability of microbial nutrients, it is tempting to speculate that LCN2 might shape the composition of the microbiome. Since the microbiome can strongly influence many aspects of the host immune system [11], this could be a major relay mediating LCN2-related immunomodulatory effects.

While immune responses need to be of sufficient intensity for pathogen clearance, inflammatory responses can cause substantial damage to the host. Indeed, certain pulmonary viral infections, including influenza and SARS-CoV-2 [12,13], are associated with particularly severe degrees of inflammation, which, considering the delicate architecture of lung tissue, require complex and efficient regulatory mechanisms [14]. Immunopathology during viral infections can be caused by both innate and adaptive immune activity, for instance by dysregulated production of proinflammatory cytokines, or exuberant cytolytic activity of CD8^+^ T cells [15]. Pulmonary dendritic cells (DCs) represent the interface between the innate and adaptive immune systems in the lungs by uptake, processing and presentation of antigens leading to initiation of specific T cell responses. In particular, CD103^+^ DCs contribute substantially to the cytotoxic T lymphocyte response during influenza infection [16]. Potential effects of LCN2 on DCs could consequently influence adaptive anti-viral immune responses. Here, we aimed to examine the role of LCN2 as modulator of lung immunity in the context of homeostasis and viral infections. Since previous studies [7–10] suggested an immunomodulatory effect of LCN2 on myeloid cells, we focused our studies on alveolar macrophages and CD103^+^ dendritic cells.

## Results

### Lipocalin 2 shapes the transcriptome of pulmonary myeloid cells during homeostasis

**Fig 1:**
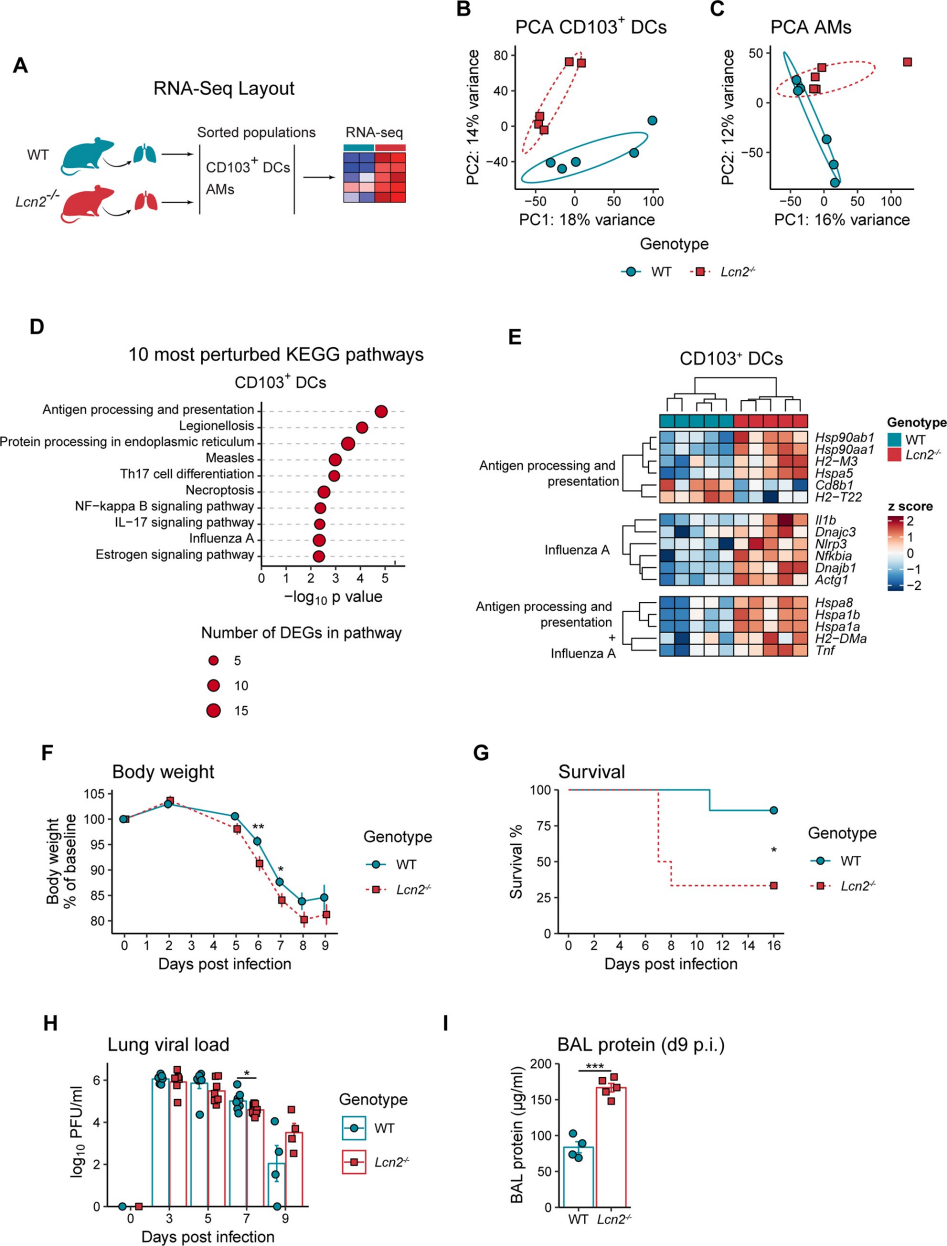
LCN2 shapes myeloid cell transcriptome during homeostasis and limits disease morbidity during influenza infection. (A) Experimental layout to assess the influence of LCN2 on the transcriptome of pulmonary myeloid immune cells. CD103^+^ dendritic cells (DCs) and alveolar macrophages (AMs) were isolated by FACS and processed for RNA sequencing. (B-C) Principal component analysis of CD103^+^ DCs (B) and AMs (C) derived from lungs of WT and *Lcn2*^*-/-*^ mice. (D) Top ten KEGG pathways with lowest SPIA (Signaling Pathway Impact Analysis) p values in CD103^+^ DCs. Circle sizes indicate the number of differentially expressed genes (DEGs) associated with the respective pathway. (E) Heatmap of DEGs in *antigen processing and presentation* or *Influenza A* KEGG pathways. Raw counts are *rlog* transformed followed by z-score scaling. (F) Comparison of relative body weight (normalized to baseline) between WT and *Lcn2*^*-/-*^ mice after infection with 10 PFU PR/8, shown as group means +/- SEM. Pooled data from three independent experiments are shown, n = 17 mice per genotype. (G) Survival of WT and *Lcn2*^*-/*-^ mice after infection with 15 PFU PR/8. n = 7 (WT) and 6 (*Lcn2*^*-/-*^). Data are representative of two independent experiments. (H) Viral load, assessed by plaque assay on MDCK cells, in lung homogenates of WT or *Lcn2*^*-/-*^ mice after infection with 10 PFU PR/8. n = 4–10 mice per genotype and timepoint. (I) Total protein concentration in bronchoalveolar lavage (BAL) 9 days after infection with 10 PFU PR/8. n = 4 (WT) and 5 (*Lcn2*^*-/-*^). Data are representative of two independent experiments. (E) Columns represent samples collected from individual mice. Bar diagrams (H, I) show group means +/- SEM. Significance was assessed using Student’s t test for (F), (H) and (I), or asymptotic two-sample logrank test for (G). *p < 0.05, **p < 0.01, ***p < 0.001.

To assess if LCN2 exerts any effects on the homeostatic transcriptome of lung-resident myeloid immune cells that could impact acute immune responses [17], we performed RNA sequencing of pulmonary CD103^+^ DCs and alveolar macrophages (AMs) isolated from wild type (WT) and *Lcn2*^*-/-*^ mice (Fig 1A). Using principal component analysis on all expressed genes, we revealed a clear separation of CD103^+^ DCs according to genotype (Fig 1B), and a tendency for AM samples (Fig 1C). Differential gene expression analysis identified 56 upregulated and 54 downregulated genes in *Lcn2*^*-/-*^ AMs (S1A Fig), and 205 upregulated and 142 downregulated genes in *Lcn2*^*-/-*^ CD103^+^ DCs (S1B Fig). While showing little overlap with AMs (S1C Fig), the LCN2-related differentially expressed genes of CD103^+^ DCs mapped to different KEGG (Kyoto Encyclopedia of Genes and Genomes) pathways (Fig 1D and S1 Table) see S1D Fig and S2 Table for differentially impacted KEGG pathways in AMs, with *antigen processing and presentation* (based on 11 differentially expressed genes) affected the most. Remarkably, three pathways related to infections (*Legionellosis* [9 genes], *Measles* [11 genes] and *Influenza A* [11 genes]), were among the 10 most significantly impacted (Fig 1D). Focusing on *antigen processing and presentation* and *Influenza A*, we identified three heat-shock proteins (*Hspa8*, *Hspa1b*, *Hspa1a*), the M alpha chain of the MHC-II complex (*H2-DMa*) and *Tnf* (Fig 1E) to be differentially regulated. The genes related to *antigen processing and presentation* further included the nonclassical MHC class I molecule H2-M3, highlighting that LCN2 affected expression of both, MHC class I and II associated genes. Interestingly, the *Influenza A* pathway was related to six additional differentially regulated genes, including *Il1b* and *Nlrp3*. These results reveal that LCN2 shapes pulmonary CD103^+^ DC gene expression profiles in homeostasis and suggest potential effects of LCN2 on antigen processing and presentation by DCs in the context of antiviral immune responses.

### Lipocalin 2-deficient animals show increased disease morbidity and lung T cell numbers upon influenza infection

Considering the importance of CD103^+^ DC in cross presenting antigen during influenza infection [16], and the obvious impact LCN2 has on homeostatic CD103^+^ DC signatures, we tested the functional implications of these findings. Using a mouse model of influenza infection based on intranasal inoculation with PR/8 (a mouse-adapted H1N1 influenza strain [18]) we determined the potential functions of LCN2 in antiviral immunity. LCN2 deficiency was associated with increased body weight loss over nine days after infection (Fig 1F), which led to higher mortality in *Lcn2*^*-/-*^ mice (Fig 1G). Interestingly, this impaired disease phenotype was not associated with increased lung viral loads in *Lcn2*^*-/-*^ mice (Figs 1H and S1E). In contrast, LCN2 deficiency was associated with increased bronchoalveolar lavage (BAL) levels of proinflammatory cytokines (i.e. IL12, interferon [IFN]γ, IP-10, IL6, MCP-1, BAFF and IFNα) three days after influenza infection (S1F Fig). Furthermore, *Lcn2*^*-/-*^ animals showed higher BAL protein levels at the peak of inflammation (nine days after influenza infection; Fig 1I), indicating increased lung vascular permeability and inflammation [19,20]. We conclude from these results that LCN2 attenuated influenza infection-related inflammation and disease morbidity independent of viral clearance, suggesting a role for LCN2 in limiting virus-related immunopathology.

**Fig 2:**
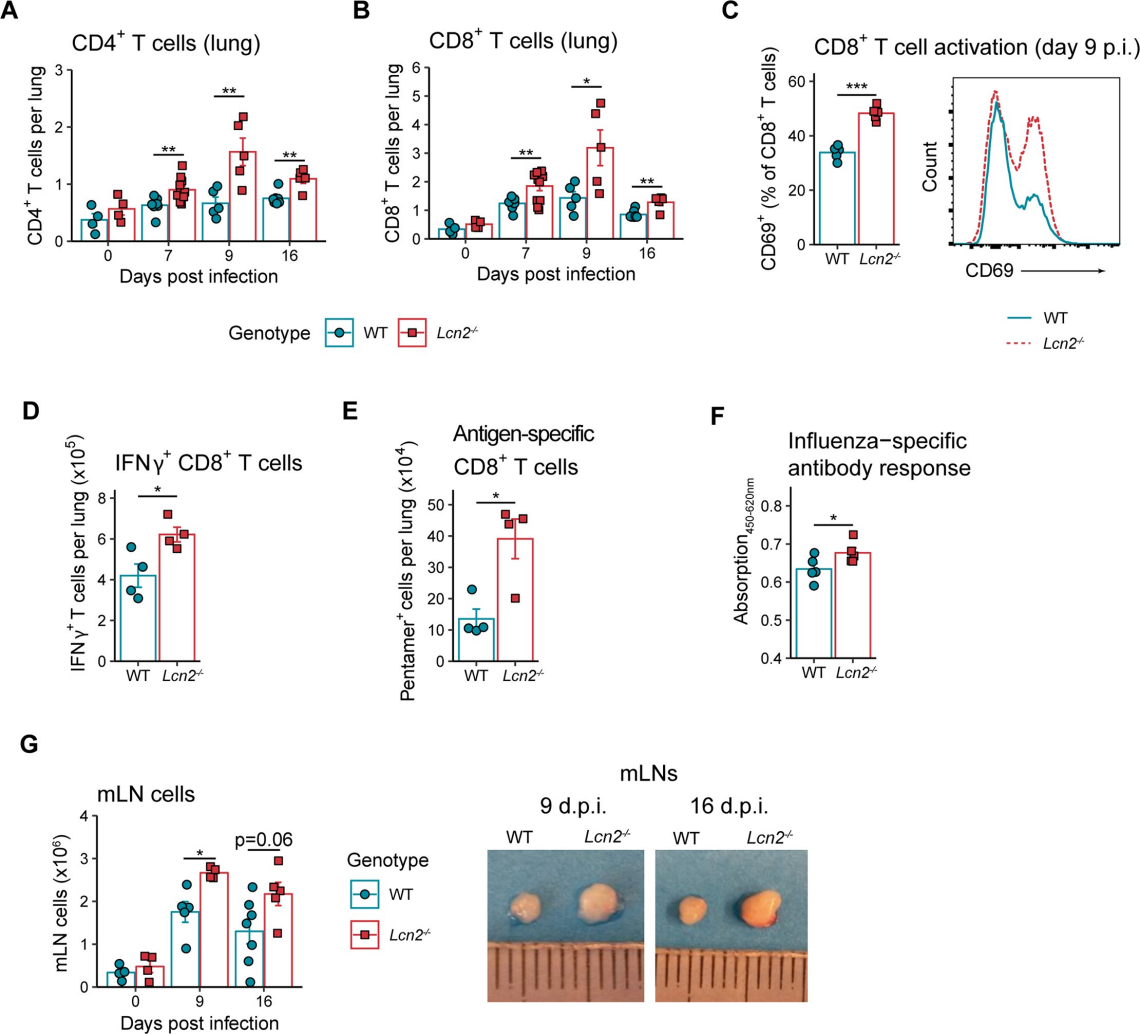
LCN2 reduces lung T cell numbers and mediastinal lymph node size during influenza infection. (A, B) CD4^+^ (A) and CD8^+^ T cell counts (B) in lungs of WT and *Lcn2*^*-/-*^ mice at baseline (d0) or at indicated timepoints after infection with 10 PFU PR/8. n = 4 (baseline), 7–10 (day 7), 5 (day 9) and 5–7 (day 16 post infection) per genotype. Data are representative of two independent experiments. (C) Percent of activated (CD69^+^) CD8^+^ lung T cells 9 days post infection. Quantification and representative histograms of are shown. n = 5 per genotype. (D) Number of IFN***γ***^***+***^ lung CD8^+^ T cells. *n = 4 per genotype*. *(E)* Number of antigen-specific (PR8 pentamer^+^) lung CD8^+^ T cells. *n = 4 per genotype*. (F) Total influenza-specific IgG levels in serum collected from mice 16 days after influenza infection. n = 5 per genotype. (G) Mediastinal lymph node (mLN) leukocyte counts and representative images of mLNs 9 and 16 days after influenza infection. n = 4 (baseline), 4–5 (day 9 post infection) and 5–7 (day 16 post infection) per genotype. Bar diagrams show group means +/- SEM. Statistical significance for comparisons between genotypes for was assessed using Student’s t test. *p < 0.05, **p < 0.01, ***p < 0.001.

**Fig 3:**
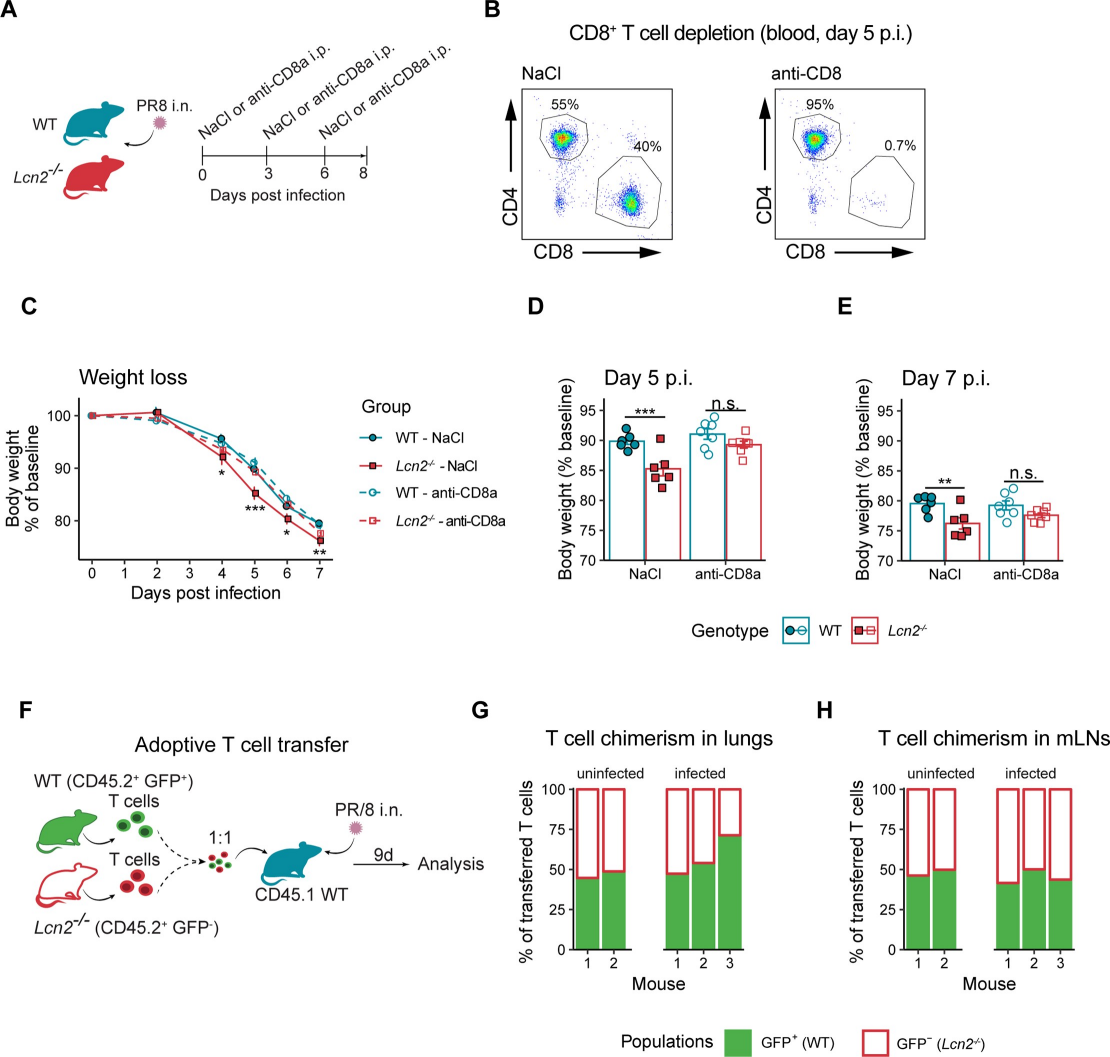
LCN2 protects from excessive weight loss by reducing lung CD8^+^ T cell numbers but does not affect T cell intrinsic proliferation potential. (A) Experimental layout to assess the contribution of CD8^+^ T cells to LCN2-related effects on weight loss during influenza infection. WT and *Lcn2*^*-/-*^ mice were treated with anti-CD8a or vehicle (NaCl) on day 0, 3 and 6 post influenza infection (p.i.). Weight loss was monitored and the experiment was stopped meeting pre-defined endpoint criteria. (B) Representative FACS plots illustrating CD8^+^ T cell depletion after anti-CD8a treatment on day 5 post infection/start of treatment. Cells are pre-gated single, live, CD45^+^, CD19^-^ and CD3^+^ cells. (C) Weight curves after influenza infection. Asterisks below the curves denote statistical significance for the comparison between NaCl-treated WT *vs*. *Lcn2*^*-/-*^ mice, asterisks above the curve for the comparison between anti-CD8a treated mice. n = 6–7 per group (D, E) Relative body weight (compared to baseline day 0) on day 5 (D) and day 7 (E) after influenza infection. (F) Experimental layout to assess the proliferative potential of WT and *Lcn2*^*-/-*^ T cells. 50:50 ratio of splenic WT (GFP^+^) and *Lcn2*^*-/-*^ (GFP^-^) T cells were transferred to CD45.1^+^ mice and infected with PR/8. CD45.2^+^ GFP^+^ and CD45.2^+^ GFP^-^ T cells (indicative of transferred WT or *Lcn2*^*-/-*^ genotype, respectively) were assessed nine days later in lungs and mesenteric lymph nodes (mLNs). (G, H) Relative abundance of GFP^+^ (WT) and GFP^-^ (*Lcn2*^*-/-*^) cells among CD45.2^+^ CD3^+^ T cells derived from lungs (G) and mLNs (H) 9 days post infection. Columns represent samples collected from individual mice. Bar diagrams and error bars (C-E) show group means +/- SEM. Statistical significance for comparisons between genotypes for (C-E) was assessed using estimated marginal mean comparison after 2-way ANOVA. *p < 0.05, **p < 0.01, ***p < 0.001.

To characterize the regulatory function of LCN2 on immunological processes during influenza infection, we analyzed the influenza-induced lung T cell response by flow cytometry (S2A Fig). We observed more CD4^+^ T helper cells (Fig 2A) and CD8^+^ cytotoxic T cells (Fig 2B) in lungs of *Lcn2*^*-/-*^ mice seven, nine and 16 days after infection. Furthermore, LCN2 deficiency was associated with elevated proportions of CD69^+^ activated pulmonary CD4^+^ (S2B Fig) and CD8^+^ T cells (Fig 2C) and higher numbers of IFNγ-expressing (Fig 2D) and of antigen-specific CD8^+^ T cells (Fig 2E). This enhanced adaptive immune response was further reflected by increased influenza-specific serum IgG antibody levels in *Lcn2*^*-/-*^ mice (Fig 2F). Apart from an increase in lung neutrophils (day nine) and B cells (day 16 after infection) in *Lcn2*^*-/-*^ mice, we observed no significant differences in the composition of lung immune cells (S2C–S2E Fig). However, the enlarged mediastinal lymph nodes (mLNs), which are considered the primary sites of early T cell proliferation after influenza infection [21], and increased mLN cell numbers nine and 16 days after influenza infection underline the increased adaptive immune response in *Lcn2*^*-/-*^ animals (Fig 2G). We next depleted CD8^+^ T cells to assess their contribution to the increased morbidity of *Lcn2*^*-/-*^. This depletion protected *Lcn2*^*-/-*^ mice from exaggerated weight loss between day 4 and 7 post infection (Fig 3C–3E), indicating that CD8^+^ T cells mediate the increased weight loss during this time period. Taken together, these results suggested that LCN2 influenced disease morbidity during influenza infection by regulation of T cell-related adaptive immunity.

### LCN2 alters the antigen presentation efficiency of CD103^+^ DCs, but not intrinsic T cell proliferation

We next aimed to determine whether the increased lung T cell numbers in *Lcn2*^*-/-*^ mice were related to an increased intrinsic T cell proliferation potential. To do so, we applied a competitive T cell chimera tracing strategy based on transfer of equivalent numbers of CD45.2^+^ splenic T cells isolated from green fluorescent protein (GFP) expressing WT mice and GFP^-^ *Lcn2*^*-/-*^ mice into CD45.1^+^ WT recipients, and subsequent influenza infection (2 days after transfer, Fig 3F). Nine days after influenza infection of engrafted CD45.1^+^ mice, we observed comparable numbers of CD45.2^+^ GFP^+^ (WT) and GFP^-^ (*Lcn2*^*-/-*^) lung (Fig 3G) and mLN (Fig 3H) T cells. These results prove that LCN2 does not influence the intrinsic proliferative potential of T cells.

**Fig 4:**
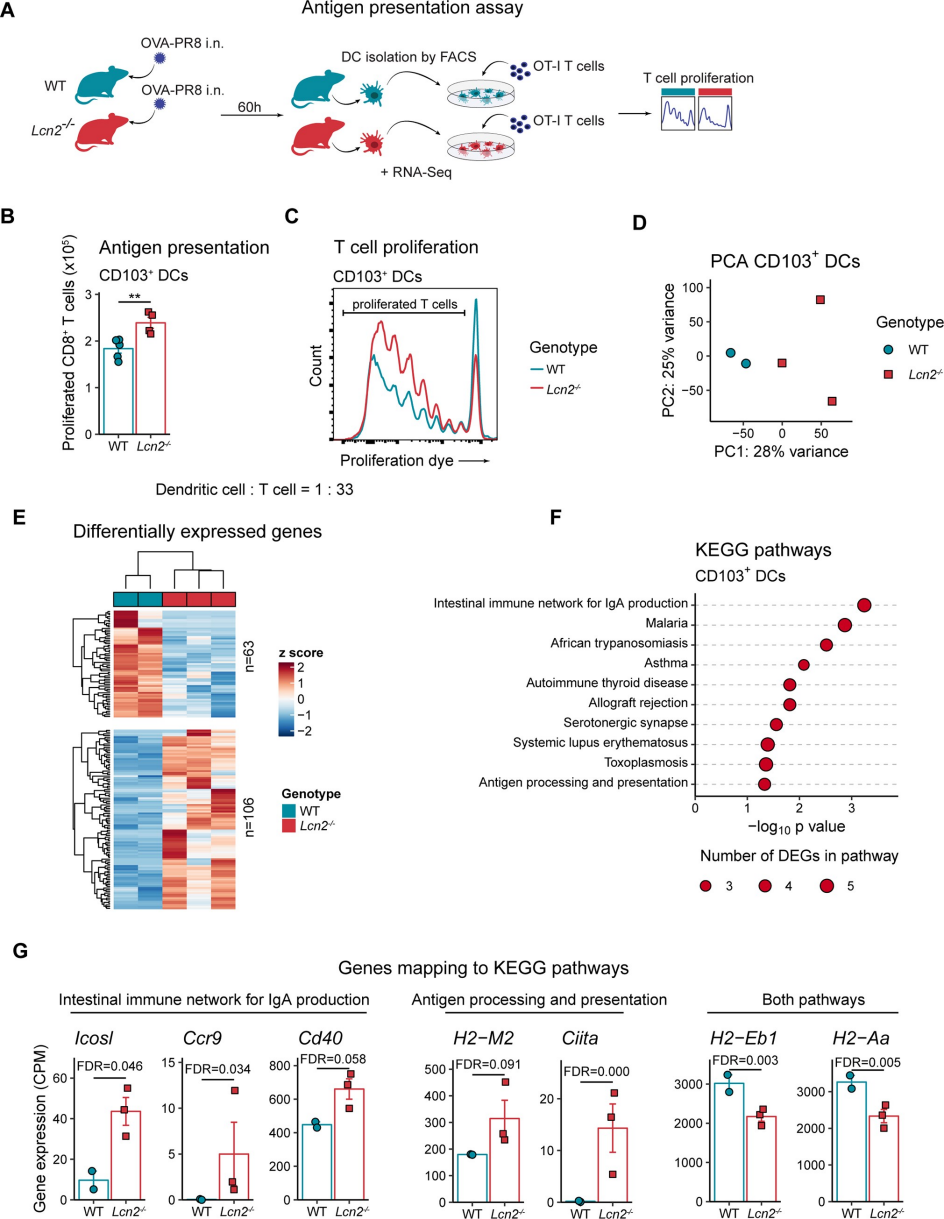
*Lcn2*^*-/-*^ CD103^+^ DCs are more proficient in presenting antigen to CD8^+^ T cells and have an altered transcriptome after influenza infection. (A) Experimental layout for antigen presentation assay. CD103^+^ and CD8^+^ DCs were sorted from mediastinal lymph nodes (mLNs) of WT and *Lcn2*^*-/-*^ mice 60h after PR/8-OVA infection, followed by co-culture with splenic OT-I T cells. (B) CD8^+^ T cell proliferation of 96h co-culture with WT or *Lcn2*^*-/-*^ CD103^+^ DCs at 1: 33 (DC: T cell) ratio. Technical replicates (n = 2–4) of two pools (pooled DCs from 6–7 mice per genotype) are shown (data of CD8^+^ DCs are in S3 Fig). (C) Representative histograms of (B). (D) Principal component analysis of transcriptomic profiles of mLN-derived CD103^+^ DCs from WT and *Lcn2*^*-/-*^ mice 60 hours after infection with PR/8-OVA. (E) Heatmap of all differentially expressed genes (DEGs) in CD103^+^ DCs. Read counts are *rlog* transformed, followed by z-score scaling. (F) Top ten KEGG pathways with lowest SPIA (Signaling Pathway Impact Analysis) p values in CD103^+^ DCs isolated from mLNs of PR/8-OVA-infected mice. Circle sizes indicate the number of DEGs associated with the respective pathway. (G) Normalized expression (counts per million mapped reads) of differentially expressed genes (FDR < 0.1) mapping to either *intestinal immune network for IgA production* or *antigen processing and presentation*, or both KEGG pathways. Bar diagrams (B), (G) show group means +/- SEM. Statistical significance for (B) was assessed using nested ANOVA. *p < 0.05, **p < 0.01.

Another reason for the increased T cell numbers in *Lcn2*^*-/-*^ mice could be an altered antigen-presentation process. The proliferation of CD8^+^ T cells during influenza infection is driven by cross-presentation of viral antigens by lung-resident DCs, which migrate from the infected lung to the mLNs [16]. To compare the potential of WT and *Lcn2*^*-/-*^ DCs to induce CD8^+^ T cell proliferation, we used an antigen presentation assay utilizing a genetically modified PR/8 strain expressing an ovalbumin peptide (PR/8-Ova) [22] in combination with OT-I CD8^+^ T cells (derived from mice which possess exclusively ovalbumin-specific CD8^+^ T cells [23]). Sixty hours after infection with PR/8-Ova, CD103^+^ DCs and CD8^+^ DCs were sorted from mediastinal lymph nodes and then incubated with OT-I T cells (Fig 4A). Using this approach, we observed increased CD8^+^ T cell proliferation upon incubation with *Lcn2*^*-/-*^ CD103^+^ DCs (Fig 4B and 4C). This LCN2-related effect was limited to CD103^+^ DCs, which are considered the dominant antigen cross-presenting DCs in the lung [16,24,25], and was not observed for CD8a^+^ DCs (S3A and S3B Fig). To further dissect the effect of LCN2 on DCs in antiviral immunity, we compared the transcriptome of WT and *Lcn2*^*-/-*^ CD103^+^ DCs collected from mLNs 60 hours after influenza infection and observed separation of samples according to genotype, suggesting robust LCN2 effects on the CD103^+^ DC transcriptome during infection (Fig 4D). Among 106 upregulated and 63 downregulated genes in *Lcn2*^*-/-*^ CD103^+^ DCs (Fig 4E), only very few (one and four genes, respectively) showed similar regulation in cells isolated from homeostatic lungs (S3D Fig), indicating specific differentially regulated gene sets during homeostasis and infection. The most significantly impacted KEGG pathway in CD103^+^ DCs isolated from infected *Lcn2*^*-/-*^ mice was *intestinal immune network for IgA production* (Fig 4F). Differentially expressed genes mapping to this pathway included *Ccr9*, *Icosl* and *Cd40*, the latter two of which are involved in interactions between DCs and T cells [26,27], and *H2−Eb1* and *H2−Aa* which encode subunits of the MHC II complex (Fig 4G). These two genes were also associated with the KEGG pathway *antigen processing and presentation*, which was also significantly affected (Fig 4F and 4G). Further genes linked to this pathway were *H2-M2*, which encodes an MHC class Ib antigen, and *Ciita*, a transcriptional coactivator that regulates MHC class I and II genes [28]. Taken together, these results show that LCN2 negatively regulates antigen presentation of CD103^+^ DCs to CD8^+^ T cells during influenza infection, possibly by influencing the expression of specific functionally important gene sets.

### The microbiome is shaped by LCN2 and influences the immune response to influenza infection

**Fig 5:**
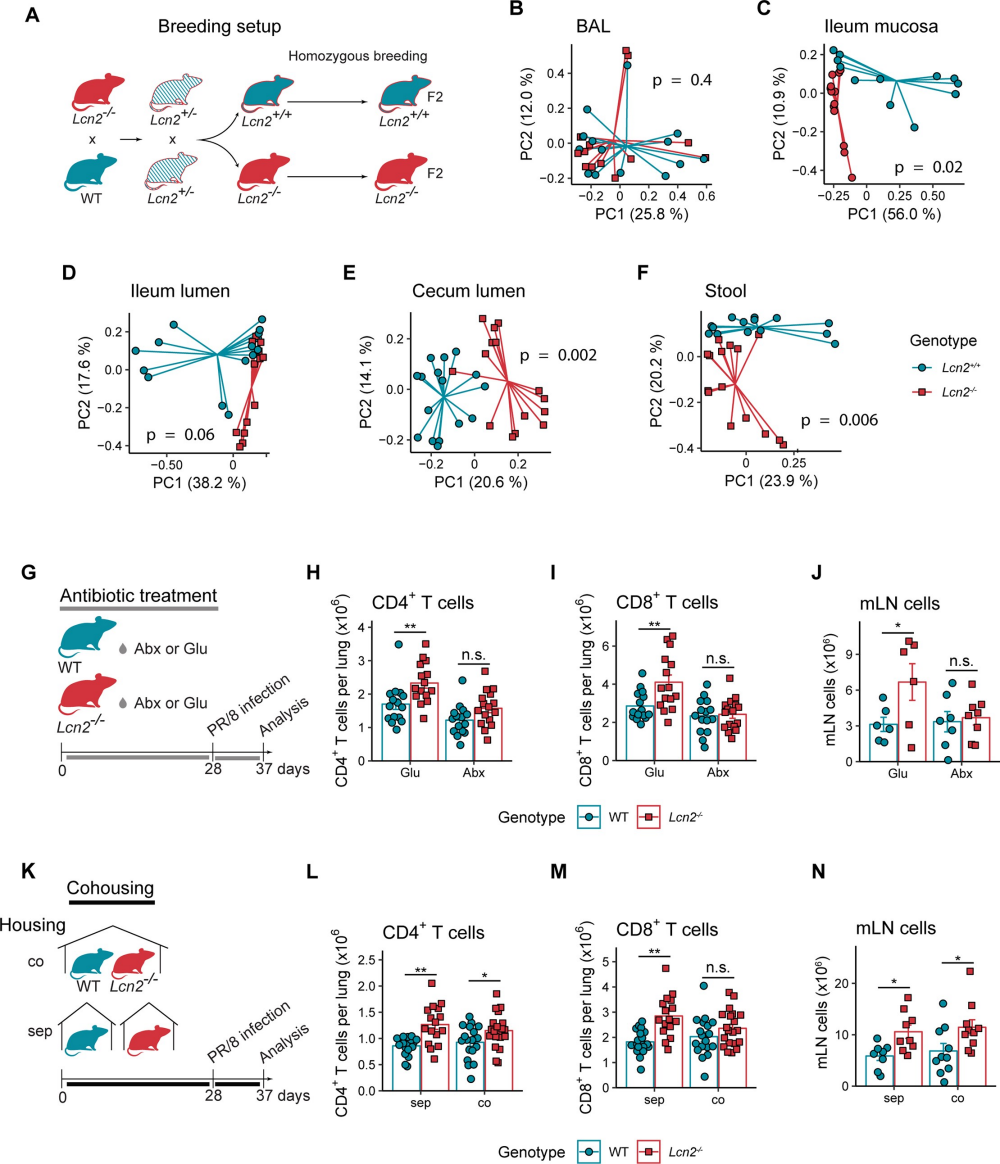
Effect of LCN2 on antiviral immunity is dependent on the microbiome. (A) Breeding setup for microbiome analysis of WT *or Lcn2*^*-/-*^ mice. (B-F) Principal coordinate analyses on Bray-Curtis distances (with PERMANOVA p-values for genotype, controlling for cage) for bronchoalveolar lavage (BAL), ileal mucosa, ileal lumen, cecum and stool samples derived from 12 weeks old WT and *Lcn2*^-/-^ mice. n = 13–16 mice per genotype. (G) Experimental setup for experiments involving antibiotic treatment prior to influenza infection. (H-J) Lung CD4^+^ T cells (H), CD8^+^ T cells (I) and total mediastinal lymph node (mLN) cell counts (J) 9 days after PR/8 infection for WT and *Lcn2*^*-/-*^ antibiotics-treated and control mice. Data from two pooled experiments are shown in (H-I). Total n per genotype and treatment = 15–16 for (H-I) or 6–7 (J). (K) Experimental setup for co-housing experiments. WT and *Lcn2*^-/-^ mice were separately- (sep) or co-housed (co) for four weeks prior to influenza infection. (L-N) Lung CD4^+^ T (L) and CD8^+^ T cell counts (M) and total mLN cell counts (N) 9 days after PR/8 infection for co- or separately-housed WT and *Lcn2*^*-/*-^. Data from two pooled experiments are shown in (L-M), total n per genotype and treatment = 16–20 for (L-M) or 8–10 (N). Bar diagrams show group means +/- SEM, and statistical significance for comparisons between genotypes for (H-J) and (L-N) was assessed using estimated marginal mean comparison after 2-way ANOVA. n.s. not significant, *p < 0.05, **p < 0.01.

Having shown that LCN2 protects from influenza-associated disease severity by modulating DC-driven T cell activation during infection, we wanted to investigate potential mechanisms that could link those observations. To this end, we generated DCs from bone marrow (BMDCs) of WT and *Lcn2*^*-/-*^ mice and investigated their antigen presentation potential *in vitro*. Interestingly, ovalbumin-pulsed WT and LCN2-deficient BMDCs were similarly proficient inducers of OT-I CD8^+^ T cell proliferation (S3D Fig). Additionally, pre-incubation of BMDC with recombinant LCN2 during antigen pulsing did not alter the antigen presentation capacity (S3E Fig). In line with these results, we considered direct effects of LCN2 on DC antigen presentation rather unlikely, and decided to explore potential indirect mechanisms. Since it is known that LCN2 modulates the availability of microbial nutrients [4] and that the microbiome can influence antiviral immunity [29,30], we tested if LCN2 availability might alter the microbiome. We therefore compared commensal intestinal and pulmonary bacterial communities in WT and *Lcn2*^*-/-*^ mice. While microbiome analysis of F2 generation offspring of heterozygous breeding pairs by 16S rRNA gene sequencing (Fig 5A) showed that the BAL microbial profile of WT and *Lcn2*^*-/-*^ animals was similar, we observed highly different LCN2-dependent compositions of ileum luminal and mucosal, cecal and stool microbial communities (Fig 5B–5F). Shannon diversity was comparable between genotypes for all sampled sites, but we observed trends towards decreased amplicon sequencing variant (ASV) richness in cecal and stool samples (S4A and S4B Fig). The bacterial phyla *Proteobacteria*, *Bacteroidetes* and *Actinobacteria* were increased in stool samples from *Lcn2*^*-/-*^ mice, whereas *Tenericutes* were decreased (S4C Fig). While phylum level differences in microbial composition were limited to stool samples, we observed differences for multiple ASVs for all sample sites throughout the intestinal tract (S4D Fig and S3 Table). As such, an ASV identified as segmented filamentous bacteria (SFB), which accounted for up to 90% of all bacterial sequencing reads in the ileal mucosa of WT mice, was absent in *Lcn2*^*-/-*^ mice (S4D Fig). In summary, our data show that LCN2 shapes the intestinal microbiome in a site-specific manner.

We next aimed to determine whether the LCN2-related microbiome differences play a role in the dysregulated antiviral immune response in *Lcn2*^*-/-*^ mice. To deplete the intestinal microbiome, we treated WT and *Lcn2*^*-/-*^ mice with antibiotics (vancomycin, metronidazole, ampicillin and gentamicin) in glucose-supplemented drinking water for 4 weeks prior to influenza infection (Fig 5G). While mock-treated *Lcn2*^*-/-*^ mice (receiving glucose-supplemented water) exhibited increased pulmonary T cell and mediastinal lymph node cell counts as previously observed, microbiome depletion equalized these differences to WT mice (Fig 5H–5J). In addition, antibiotic treatment abolished differences in the antigen presentation potential of *Lcn2*^-/-^ and WT CD103^+^ DCs (S5A and S5B Fig). To further study the role of the microbiome, we co-housed WT and *Lcn2*^*-/-*^ mice for 4 weeks prior to PR/8 infection (Fig 5K) to partially hybridize microbial profiles by passive transfer of microbiota [31,32]. Confirming the effectivity of this approach, co-housing led to similar levels of the bacterial phylum *Bacteroidetes* in WT and *Lcn2*^-/-^ animals, while significantly different in separately housed mice (S4C Fig). Finally, co-housing decreased lung CD8^+^ T cell differences between influenza-infected WT and *Lcn2*^*-/-*^ mice (Fig 5L and 5M), while mLN size was unaffected (Fig 5N). Taken together, these results indicate that LCN2 modulated the antiviral immune response and prevented exaggerated CD8^+^ T cell immunity during influenza infection in a microbiome-dependent mechanism.

## Discussion

Previous studies provided evidence for the immunomodulatory potential of LCN2 [7–10]. We here extend and substantiate these observations by showing that LCN2 skewed the transcriptome of lung myeloid cells during homeostasis and dampened T cell responses upon influenza infection, resulting in diminished disease-related morbidity and mortality independent of viral clearance. We succeeded in linking the effects of LCN2 to the magnitude of CD8^+^ T cell responses via altered CD103^+^ DC functionalities. Finally, the LCN2-driven immunomodulation did not occur directly, but relied on LCN2’s impact on the microbiome composition, which we verified by experiments using antibiotics or cohousing approaches.

Influenza infection-related pathology in humans, non-human primates and mice is predominantly caused by exaggerated antiviral and hyper-inflammatory responses, rather than direct cytopathic effects of the virus [12,33–35]. While critical for viral clearance and immunological memory, pro-inflammatory cytokine production and cytolytic activity by CD4^+^ and CD8^+^ T cells, respectively, importantly contribute to immunopathology [36,37]. Our data indicate that excessive T cell proliferation, resulting from more potent antigen presentation by CD103^+^ DCs to CD8^+^ T cells, mediate increased morbidity in *Lcn2*^*-/-*^ mice. Our study identifies LCN2 as an important modulator of antiviral immunity and suggests that LCN2 fine-tunes DC activity during homeostasis and to prevent excessive T cell expansion and immunopathology during viral infections. Of note, tissue damage due to aberrant immune activation is not restricted to influenza infections, but has been suggested to play an important role in COVID-19 mortality [13]. While we here focused on influenza infection, LCN2-regulated inflammation and immunopathology might also importantly influence the course of other viral infections [15].

Recent research highlights the critical effect of microbiome compositions on maturation and functionality of the immune system [11]. As such, the gut microbiome was reported to influence DC migration to draining lymph nodes and T cell priming during influenza infection by providing signals leading to pro-IL1β and pro-IL18 expression at steady state and inflammasome activation upon influenza infection [29]. While antibiotic treatment interfered with these signals, intranasal or intrarectal injection of TLR ligands was sufficient to restore the microbiome dependent effects. Furthermore, sensing of host microbiota via TLR5 was found to facilitate antibody responses to influenza vaccines by promoting plasma cell differentiation [38]. Additionally, microbial metabolites such as desaminotyrosine and short-chain fatty acids were discovered to regulate antiviral immunity and to protect from influenza associated disease morbidity [39,40]. We here observed that LCN2-deficiency was associated with an altered site-specific intestinal microbial composition and that microbiome disruption by antibiotics abrogated LCN2-related differences in anti-influenza immunity. While we did not identify which specific features of the microbiota mediated the observed effects on host immunity, we identified several candidates. Among the multiple ASVs with differential abundance in ileal, cecal or stool samples, SFB (which were among the most abundant ASVs in the ileal mucosa of WT mice) were completely absent in *Lcn2*^*-/-*^ mice. This common bacterial strain is involved in host-microbiome crosstalk as it can induce Th17 cells in the gut [41], and its abundance is affected by influenza infection [42].

Two previous studies have set out to assess the effect of LCN2 on stool microbial composition. Singh *et al*. reported distinct bacterial communities in *Lcn2*-deficient mice, which were linked to exacerbated colitis or neutralization of IL10 [43]. In agreement with our findings, this study found increased *Bacteroidetes* and *Proteobacteria* phyla and a decreased *Tenericutes* phylum in stool samples of *Lcn2*^*-/-*^ mice. In contrast, Moschen *et al*. reported that LCN2 exclusively affects the intestinal microbiome in mice lacking IL-10 leading to colitis and spontaneous emergence of right sided colonic tumors [44]. These discrepancies could be explained by altered baseline microbiota due to differences in animal housing or the lower number of experimental animals with differences in statistical power in the study by Moschen *et al*. (4 *vs* 13–16 mice per genotype in the present study).

Several cell surface receptors for LCN2 have been identified: both SLC22A17 (also named 24p3R) and megalin bind LCN2 and mediate its cellular uptake [45,46]. Furthermore, LCN2 can bind to and signal through MC4R in the hypothalamus [3]. We conclude from our results that the effects of LCN2 on influenza-associated T cell responses are primarily dependent on the microbiome. While LCN2 exposure of DCs did not alter their antigen presentation potential *in vitro*, we cannot completely rule out that direct effects of LCN2 on immune cells contribute to the immune-regulatory properties of LCN2 *in vivo*. Further research is needed to fully elucidate these mechanisms. Considering its drastic immunomodulatory effects, variable LCN2 levels in human subjects [47] could have important clinical consequences. For instance, decreased LCN2 levels might identify patients at risk of exaggerated responses to inflammatory triggers such as viral infections, and, on the other hand, supraphysiological LCN2 levels might prevent proper function of the adaptive immune system. Recent evidence suggests a beneficial effect of chronically increased LCN2 levels in patients with obesity or type 2 diabetes [48,49], as it assists in counteracting obesity-induced glucose intolerance by reducing appetite and driving beta-cell proliferation [50]. Interestingly, diabetes and obesity are also associated with increased susceptibility to severe influenza infection [51,52], but the potential correlation between LCN2 levels and disease outcome in humans is not known. Considering the dampening effects of LCN2 on adaptive immunity and influenza-specific antibody levels, elevated LCN2 levels could be a reason for the poor vaccination responses observed in obese individuals [53]. While these observations can be interpreted as potential therapeutic scenarios, further research is required to test the applicability and translational feasibility of such LCN2-related treatment strategies.

## Materials and methods

### Ethics statement

All mouse experiments were performed in accordance with Austrian law after approval by the Austrian Ministry of Sciences (protocol ID BMWFW-66.009/0285_WF/V/3b/2014 and BMBWF-66.009/0084-V/3b/2018).

### Mice

Experiments were conducted using specific opportunistic pathogen free (SOPF) age-matched 8- to 12-week-old C57BL/6J WT, *Lcn2*^*–/–*^[4], CD45.1 [54] and B6-GFP mice [55]. *Lcn2*^*-/-*^ mice were provided by S. Akira (University of Osaka, Osaka, Japan), and backcrossed to C57BL/6J mice for 10 generations. OT-I transgenic (C57BL/6-Tg(TcraTcrb)1100Mfb/J)) mice [23] were obtained from Maria Sibilia (Medical University of Vienna). Apart from survival experiments, which were conducted in male mice, all experimental procedures were carried out in female mice. To analyze the effect of LCN2 on the gut and lung microbiome, we utilized a breeding scheme based on recommendations by Mamantopoulos *et al*. [56]. Briefly, WT and *Lcn2*^*-/-*^ mice with a C57BL/6 background were intercrossed, generating *Lcn2*^*+/-*^ mice. By breeding these mice, we generated *Lcn2*^*+/+*^ and *Lcn2*^*-/-*^ littermates (F1 generation from heterozygous mice), which were bred separately. The offspring of these mice (F2 generation) were used for microbiome analysis.

### Influenza virus strains

Purified influenza A/PR/8/34 (PR/8) virus was obtained from Charles River, diluted 1:10 in sterile PBS, aliquoted and stored at -80°C. PR/8-OVA [22] was provided by Adolfo García-Sastre (Icahn School of Medicine at Mount Sinai, New York). Viral titers were determined by plaque forming assay using MDCK cells [57]. One day before the assay, 1x10^6^ cells/well were seeded in 6-well plates and grown overnight in Dulbecco’s Modified Eagle Medium (DMEM; Sigma), supplemented with 10% fetal calf serum (FCS; Sigma), 100 U/ml penicillin and 100 μg/ml streptomycin, to obtain monolayers with >90% confluency. Cells were then washed with PBS and serial 1:10 dilutions of virus in DMEM (without FCS and antibiotics) were added to the cells for 1 hour at 37°C (each dilution in duplicates). Inoculates were aspirated and overlay medium, consisting of DMEM with antibiotics supplemented with 0.005% DEAE-Dextran (Sigma), 0.2% endotoxin-free bovine serum albumin (Fisher Scientific), 0.2% TPCK-treated trypsin (Sigma) and 1% agarose (Biozym), was added. 3 days later, plaques were visualized by staining with 0.03% neutral red (Sigma) for 3 hours and quantified.

### Mouse model of influenza infection

For influenza infection, female mice were intranasally infected with 10–12.5 PFU PR/8 in 50 μl sterile injection-grade (sterile and endotoxin-free) 0.9% NaCl solution. For survival experiments, male mice received 15 PFU PR/8 in 50 μl. Mice were monitored daily and graded according to an internally developed scoring sheet, which assessed appearance, posture, change in body weight and body temperature, natural behavior and clinical signs. Animals with a combined score of >5 or individual scores (in one of the categories) of >3, which could result from hunched posture, more than 30% body weight loss, >5 degrees of body temperature drop, squeaking, self-mutilation or inactive behavior and visible signs of unrelated infections or bleeding, were euthanized. In some planned end-point experiments, bronchoalveolar lavage was performed by inserting a tracheal cannula (Venflon, BD Bioscience) and flushing the lungs with 1 ml NaCl. Otherwise, lungs were flushed by injecting 5 ml of endotoxin-free PBS (Gibco) into the left ventricle, removed under sterile conditions and processed. For plaque assay or RT-PCR, lung tissue was homogenized using a Precellys 24 homogenizer (Peqlab). For plaque assays, homogenates where directly stored at -80°C, whereas for RT-PCR, aliquots of lung homogenates where frozen (-20°C) in RA1 buffer (Macherey-Nagel) with 10% beta-mercaptoethanol (Calbiochem).

### Determination of viral load by qPCR

RNA was isolated using the NucleoSpin® RNA II kit (Macherey-Nagel), reverse transcription was performed using 700ng of isolated RNA and the iScript cDNA Synthesis Kit (Biorad), according to manufacturer’s protocol. Real-time PCR was performed with SYBR Green Master Mix reagents (Applied Biosystems) on a StepOnePlus Real-Time PCR System (Applied Biosystems). Primer sequences targeting the influenza M gene [58] were *CATGGAATGGCTAAAGACAAGACC* (forward) or *CCATTAAGGGCATTTTGGACA* (reverse). The reference gene HPRT was quantified using the sequences *GTTAAGCAGTACAGCCCCAAAATG* (forward) and *AAATCCAACAAAGTCTGGCCTGTA* (reverse).

### Multiplex immunoassay for measurement of BAL cytokines

BAL cytokines were measured using a custom multiplex immunoassay (eBioscience), according to the manufacturer’s protocol. Briefly, magnetic beads where incubated with undiluted BAL (performed with 1ml sterile and endotoxin-free 0.9% NaCl solution) samples in a 96-well plate for two hours, followed by washing and incubation with detection antibody (30 minutes). After washing, incubation with streptavidin-PE and another washing step, the plate was read on a Luminex 200 instrument (R&D Systems).

### Cell suspension preparation and flow cytometry

For flow cytometry analysis of lung tissue samples, representative pieces of each pulmonary lobe were weighed and homogenized using the lung dissociation kit (Miltenyi Biotec), according to the manufacturer’s protocol. Tissue was digested for 30 minutes at 37°C using DNAse I (12 U/ml; Sigma) and Collagenase I (160 U/ml; Gibco). Next, suspensions were filtered using 70 μM cell strainers (BD Biosciences), red blood cells were lysed using ACK lysis buffer (150 mM NH_4_Cl, 10 mM KHCO_3_, 0.1 mM Na_2_EDTA, pH 7.2–7.4; all chemicals from Sigma) and suspensions were again filtered through 40 μM strainers. PBS with 0.5% bovine serum albumin (BSA; BDA, cat. no. 8076.3) was used to stop red blood cell lysis and as buffer for all subsequent steps. In experiments assessing T cell antigen specificity, single cell suspensions were incubated with fluorescently labelled recombinant PR8 pentamer (ProImmune, dilution 1:5) for 10 minutes at room temperature. Cells were then incubated with anti-mouse CD16/32 antibody (TruStain fcX; BioLegend, dilution 1:500) to block unspecific binding to Fcγ receptors and viability dye (Fixable Viability Dye eFluor 780; eBioscience, dilution 1:1000) for 20 minutes at 4°C, followed by staining with fluorescence-labelled antibodies (see S4 Table, 40 minutes, 4°C). Mediastinal lymph nodes were mashed through 70 μM cell strainers, followed by Fcγ receptor blocking and staining. Cells were fixed using Fix and Perm (Nordic MUbio) reagents, according to the manufacturer’s protocol. Analysis was performed using an LSR Fortessa (BD Biosciences) and FlowJo software (FlowJo LLC). Absolute cell numbers for lungs and lymph nodes were determined using counting beads (123 count eBeads, Thermo Fisher Scientific) or a hematocytometer (for spleens).

For intracellular IFNγ staining, lung single cell suspensions were resuspended in RPMI medium containing 10% FCS (both from Sigma), 1% penicillin/streptomycin (Gibco) and 50 μM 2-Mercaptoethanol (Gibco), followed by treatment with phorbol 12-myristate 13-acetate (PMA; 100 ng/ml, Sigma), ionomycin (500 ng/ml, Sigma) and GolgiStop (1:1250, BD Biosciences) for 5 hours. Next, cells were stained for surface markers (as described above), fixed and permeabilized using BD Cytofix/Cytoperm reagents (BD Biosciences), according to manufacturer’s instructions, and stained for IFNγ using a fluorescence-labelled antibody (see S4 Table, 40 minutes, 4°C).

### Analysis of influenza specific IgG response

Influenza-specific antibodies were quantified by ELISA, whereby MaxiSorp ELISA plates (Nunc) were coated overnight at 4°C with UV-inactivated PR/8 (4000 PFU/ml). After blocking of unspecific binding (PBS 1% BSA), 50 μl of 1: 12500 diluted serum samples were added, followed by detection antibody (biotin-conjugated goat anti-mouse IgG, Jackson ImmunoResearch) and horseradish peroxidase-conjugated streptavidin (BD Pharmingen). Signal emitted by TMB liquid substrate (Sigma) was detected at 450 nm (620 nm reference) using a Sunrise plate reader (Tecan).

### CD8+ T cell depletion

To deplete CD8^+^ T cells, WT and Lcn2^-/-^ mice where intraperitoneally injected with 200 μg of anti-CD8a (clone YTS 169.4) in 200 μl NaCl on the day of infection with 12.5 PFU PR/8, and on day 3 and day 6 post infection [59]. Control mice received sterile PBS (carrier) injections on the same timepoints. Depletion of CD8^+^ T cells was assessed by FACS analysis of blood samples collected on days 2 and 5 post infection from the retro-orbital vein.

### Adoptive T cell transfer

Single cell suspensions were prepared from GFP^+^ WT and Lcn2^-/-^ (CD45.2 background for both) mouse spleens as described above, and incubated with biotinylated antibodies directed against mouse MHC-II (clone M5/114.15.2, BioLegend, diluted 1:100), B220 (clone RA3-6B2; BioLegend, diluted 1:50), CD11c (clone N418; BioLegend, diluted 1:100) and CD11b (clone M1/70, BioLegend, diluted 1:100) for 20 minutes. Spleen T cells were enriched by subsequent removal of B cells, DCs and macrophages by streptavidin magnetic beads (BD Imag system, BD Biosciences), according to the manufacturer’s protocol. The remaining enriched T cells were washed, counted with a hematocytometer, and 10^6^ T cells were injected intravenously into CD45.1 mice, followed by intranasal infection with PR/8 48 hours later. Mice were sacrificed 9 days after infection, and CD45.2^+^ GFP^+^ and GFP^-^ CD3^+^ T cells from lungs and mediastinal lymph nodes were quantified using flow cytometry.

### Bone marrow derived dendritic cells (BMDCs)

Bone marrow was isolated by flushing sterilized mouse femurs with 10 ml of RPMI medium. Bone marrow cells were cultured in tissue-culture-treated 6-well plates (Corning), in 4ml RPMI medium containing 10% FCS, 1% penicillin/streptomycin and 25 ng/ml murine GM-CSF (PeproTech), at a density of 10^6^ cells per ml. On day 2, half of the medium was replaced with fresh medium containing 25 ng/ml murine GM-CSF. On day 3, the complete medium (containing non-adherent cells) was replaced with fresh medium containing 25 ng/ml murine GM-CSF. Non-adherent cells were harvested on day 6 by gentle washing with sterile endotoxin-free PBS (Sigma) and incubated with biotinylated anti-mouse CD11c (clone N418; BioLegend). CD11c^+^ cells (BMDCs) were purified by magnetic separation using streptavidin-coated magnetic beads (IMag Cell Separation System; BD). Isolated BMDCs were pulsed with ovalbumin (Grade V, Sigma, 100 μg/ml) for 16 hours. For some experiments, recombinant LCN2 (BioLegend) was added during the pulsing step at indicated concentrations. Ovalbumin-pulsed BMDCs were used for antigen presentation assays as described below.

### Antigen-presentation assay

Mice where intranasally infected with 10000 PFU of PR/8-OVA in 50 μl. Sixty hours after infection, mediastinal lymph nodes were harvested. To maximize DC yield, lymph nodes were digested in RPMI medium supplemented with 5% FCS, DNAse I (43.3 U/ml, Sigma) and Liberase TL (12.5 μg/ml, Roche) for 25 minutes at 37°C, followed by mashing through 70 μm strainers. Obtained single cell suspensions where incubated with anti-mouse CD16/32, followed by biotinylated anti-CD3 (clone 17A2, BioLegend), anti-CD19 and anti-B220 antibodies (dilution 1:100 for each). Next, samples were incubated with streptavidin coated magnetic beads (20min, 4°C), and B and T lymphocytes were depleted using magnetic cell sorting (negative selection, BD Imag system) according to manufacturer’s instructions. The remaining cells were washed, stained with viability dye, anti-mouse CD11b-Pe-Cy7, CD8a-FITC, CD103-BV421, CD45-eVolve 605 and CD11c-PE (see S4 Table for details) and live CD103^+^ or CD8a^+^ DCs were sorted on a FACSAria Fusion cytometer (BD Biosciences). To obtain OT-I transgenic CD8^+^ T cells, single cell suspensions were prepared from spleens as described above, and incubated with biotinylated antibodies directed against mouse CD19 (clone 6D45, BioLegend), B220, CD4 (clone GK1.5; BioLegend), CD11c and Ly6G, followed by negative selection with streptavidin-coated magnetic beads (BD Imag system). The remaining enriched CD8^+^ T cell suspension was labelled using Cell Proliferation Dye eFluor 450 (eBioscience), according to the manufacturer’s protocol. Labelled OT-I transgenic CD8^+^ T cells were co-cultured with sorted DCs, at 1:100 or 1:33 DC:T cell ratios, or with ovalbumin-pulsed BMDCs (see above), 1:10 DC:T cell ratio, in RPMI medium supplemented with 10% FCS, 1% penicillin/streptomycin, 1% MEM Non-Essential Amino Acids Solution, 10 mM HEPES, 50 μM 2-mercaptoethanol and 1 mM Sodium Pyruvate (all Gibco). After 96 hours, cells were washed, labelled with fluorescent antibodies (see S4 Table) and live, CD45^+^/CD3^+^/CD8^+^/proliferation dye-low (indicating proliferation) cells were quantified by flow cytometry.

### RNA sequencing

To quantify DC or AM gene expression, 200 alveolar macrophages (defined as single/live/CD45^+^/Ly6G^-^/CD11c^+^/SiglecF^+^) or CD103^+^ DCs (defined as single/live/CD45^+^/Ly6G^-^/SiglecF^-^/F4/80^-^/CD11c^+^/MHCII^+^/CD103^+^ [lung] or single/live/CD45^+^/CD11c^+^/CD8^-^/CD11b^-^/CD103^+^ [lymph node]) were FACS-sorted from mouse lung or lymph node single cell suspensions (prepared as indicated above) into 4μ μl cell lysis buffer (nuclease-free H2O [Life Technologies, cat. no. AM9930] with 0.2% Triton X-100 [Sigma] and RNase Inhibitor [2 U/μl, Takara/Clonentech]) using a FACSAria Fusion cytometer (BD Biosciences). Cell lysates were stored at -80°C until library preparation according to the Smart-Seq2 protocol [60]. Pooled libraries were sequenced using the 50 bp single-read setup on the Illumina HiSeq 2000/2500 at the Biomedical Sequencing Facility of CeMM and the Medical University of Vienna.

### RNA sequencing data analysis

Sequencing reads were adapter-trimmed using Trimmomatic [61] and aligned to the *mm10* reference genome (*STAR aligner* [62]). Reads mapping to genes were counted using the *summarizeOverlaps* function (*Bioconductor* R package *GenomicAlignments* [63]). Differential gene expression was assessed using *DESeq2* [64], whereby separate models per cell type and condition (lung-homeostasis, lymph node-infection) were formulated for all pairwise comparisons between genotypes. Genes were filtered using independent hypothesis weighting (*ihw* R package [65]). Impact of differential expression on KEGG pathways was assessed using Signaling Pathway Impact Analysis (SPIA, *SPIA* R package [66]). Genes with an FDR-adjusted p value of < 0.1 were considered differentially expressed.

### Bacterial DNA collection, extraction, library preparation and sequencing

Sterile surgical tools were used for collection of microbial samples from mice. For lung samples, BAL was performed 10 times with 1 ml NaCl (aliquots were subsequently pooled) as described above. Next, the abdomen of the animal was sterilized using 70% EtOH and the peritoneal cavity was opened. Ileal samples were collected by cutting a 2.5 cm long piece of the terminal ileum 2 cm distal of the ileo-cecal valve. Cecal samples were collected by dissecting the whole cecum. From ileum and cecum, luminal samples were collected in a sterile microcentrifuge tube (Eppendorf Biopur) by gently squeezing out content from the distal end of the dissected intestinal segment. For collection of mucosal samples, the ileal segments where flushed with 10 ml NaCl to remove all luminal content, cut open longitudinally and scraping biopsies where collected using sterile glass slides. Bacterial DNA was isolated using the QIAamp DNA Microbiome Kit (Qiagen) according to the manufacturer’s protocol and isolated DNA was eluted in 50 μl of DEPC-treated water (Roth). To control for contaminations introduced during sample collection, two harvest controls (NaCl used to flush cannulas inserted into mouse tracheas, without performing BAL), one isolation control and one PCR control were prepared identically to BAL samples and sequenced. Similarly, another PCR control sample was prepared for ileal mucosa samples. Isolated bacterial DNA from samples and controls was amplified using barcoded, Illumina adaptor-linked PCR primers that target the V1-V2 hypervariable region of the bacterial 16S rRNA gene [67]. Each sample was amplified using the Accuprime Taq DNA Polymerase High Fidelity kit (Invitrogen) with the following cycling parameters: Initial denaturation for 2 min at 94°C, followed by amplification cycles starting with 30 sec denaturation at 94°C, 30 sec annealing at 56°C, and 60 sec elongation at 68°C, with a final extension at 68°C for 7 min. An initial enrichment PCR reaction (10 amplification cycles) was performed, followed by the amplification PCR. The number of cycles performed for amplification PCR varied between sampling sites: Stool: 20 cycles; ileum lumen: 25 cycles; cecum lumen: 35 cycles; ileum mucosa: 40 cycles. For BAL, two amplification PCRs were performed, with 40 (first reaction) and 35 (second reaction) cycles. The ideal number of amplification cycles for each sampling site was determined by increasing the number of cycles by five (starting from 20), until samples were sufficiently amplified (i.e. presence of a visible band by automated electrophoresis using an Agilent 4200 TapeStation system). All PCR reactions contained 2.5 μl of 10x AccuPrime buffer II, 0.5 μl of each 10 mM forward and reverse primers and 0.1 μl of AccuPrime Taq DNA Polymerase, to which 1 μl of template DNA and 20.4 μl of ultrapure water (for the enrichment PCR reaction) or 2 μl of the enrichment PCR reaction and 19.4 μl of ultrapure water (for the amplification PCR reaction) were added. PCR products were screened for sufficient amplification and quantified using automated electrophoresis. Finally, libraries containing pooled equimolar PCR products and spiked with 40% phiX (Illumina) were sequenced using Illumina MiSeq technology in the 2x 350bp configuration (MiSeq Reagent kit v3) at the Biomedical Sequencing Facility of CeMM and the Medical University of Vienna.

### 16S rRNA gene sequencing data processing and analysis

Raw sequences were demultiplexed using qiime [68] commands *split_libraries*.*py* with options-r 999 -n 999 -q 0 -p 0.0001 (to prevent quality filtering at this stage) and split_sequence_file_on_sample_ids.py. ASVs (amplicon sequence variants) were selected using dada2 [69]. Forward and reverse reads were trimmed to a length of 225 bp and filtered using parameters *maxN = 0*, *maxEE = c(2*,*2)* and *truncQ = 2*. Potential contaminants within unique ASVs were identified with the *decontam* R package [69] using prevalence of ASVs in control samples (for ileal mucosa and BAL samples) and correlation of ASV frequency with DNA concentration after PCR amplification as identification methods. Following contaminant removal, taxonomy was assigned against the SILVA 16s rRNA database [70] and ASVs which could not be assigned to kingdom *Bacteria* were removed from the dataset. One ASV was identified as belonging to genus *Candidatus_Arthromitus*. Since the misclassification of mammalian segmented filamentous bacteria (SFB) as *Candidatus_Arthromitus* (which would not be expected to be found in mammalian guts) is well described [71], we refer to this ASV as SFB. Next, samples with a total ASV count of less than 10000 were excluded from further analysis. The remaining samples were rarefied to the smallest library size above 10000 ASVs for the sampling site (between 10033 for stool samples and 27693 for cecal samples). Shannon diversity and Chao 1 ASV richness were calculated using the *vegan* R package [72]. Differences between genotypes (controlling for cage effects) were assessed using the *nested*.*npmanova* function (*biodiversityR* package) on ASV counts normalized to sample library size (relative abundances). Statistical comparisons of Chao1 richness and Shannon diversity were carried out with linear mixed models using the *lme4* and *lmerTest* R packages [73,74], while cage association was entered as a random factor. Linear mixed models were also used to determine which ASVs were differentially abundant according to genotype, after transforming ASV counts with a centralized log ratio (clr) transformation.

### Antibiotic treatment and cohousing

For experiments involving microbiome perturbation by antibiotics, female WT or *Lcn2*^*-/-*^ mice were treated with a mix of metronidazole (‘Kabi’, Fresenius), vancomycin (Vancocin, Baxter), ampicillin (Standacilin, Sandoz) and gentamicin (Braun, concentration of each 500 mg/l) in drinking water supplemented with 0.4% D-glucose (Sigma) starting at 5–6 weeks of age. Control mice received drinking water supplemented with 0.4% D-glucose. After 4 weeks of antibiotic treatment, mice were infected with 10 PFU PR/8. Antibiotic treatment was continued until the end of the experiment. For co-housing experiments, 4 weeks old WT or *Lcn2*^*-/-*^ mice were housed together at equal ratios for 4 weeks prior to influenza infection. Control WT or *Lcn2*^*-/-*^ mice were housed separately. As with antibiotic treatments, housing conditions were maintained throughout the infection period.

### Quantification of bacterial classes using qPCR

Bacterial DNA from stool of separately housed and co-housed WT and *Lcn2*^*-/-*^ animals was isolated using the QIAamp DNA Mini Kit (Qiagen) according to the manufacturer’s recommendations. Perfecta SYBR Green SuperMix (Quanta) on a StepOnePlus Real-Time PCR System (Applied Biosystems) were used for real-time qPCR. Primer sequences for *Bacteroidetes* were CRAACAGGATTAGATACCCT (forward) or GGTAAGGTTCCTCGCGTAT (reverse) [75]. The conserved bacterial (pan-bacteria) 16S-gene was quantified using the sequences TCCTACGGGAGGCAGCAGT (forward) and GGACTACCAGGGTATCTAATCTT (reverse) and used for calculation of log_2_ fold changes.

### Statistical analysis

Comparisons between two groups were performed using Student’s t test, except where noted otherwise. In experiments involving two grouping factors (e.g. genotype and antibiotic treatment), 2-way ANOVA was conducted, and group means were compared using the *emmeans* R package. Survival curves were compared using the asymptotic two-sample logrank test, as implemented in the *coin* R package [76]. In antigen presentation assays, technical replicates of biological replicates were analyzed. To avoid pseudo-replication, nested ANOVA was conducted in these experiments. Where appropriate, p values were corrected for multiple testing using the false-discovery rate approach [77]. Single p values below 0.05 or FDR-corrected p values below 0.1 (RNA-Seq and 16S rRNA gene sequencing) were considered statistically significant.

## Supporting information

S1 Fig Myeloid cell transcriptome and lung viral load in WT and *Lcn2*^*-/-*^ mice. (A-B) Alveolar macrophages (AMs) and CD103^+^ DCs were isolated from lungs of WT and *Lcn2*^*-/-*^ animals by FACS and prepared for RNA sequencing. Heatmaps of differentially expressed genes (DEGs) for AMs (A) and CD103^+^ DCs (B). Read counts are *rlog* transformed, followed by z-score scaling. (C) Venn diagrams illustrating overlaps in up- or down-regulated genes according to genotype between AMs and CD103^+^ DCs. (D) Significantly perturbed KEGG pathways (p value < 0.05) with lowest SPIA (Signaling Pathway Impact Analysis) p values in AMs isolated from mediastinal lymph nodes of PR/8-OVA-infected mice. Circle sizes indicate the number of DEGs associated with the respective pathway. (E) Lung viral load, as measured by qPCR, for WT and *Lcn2*^*-/-*^ mice, at indicated timepoints after PR/8 infection. (F) Heatmaps of indicated bronchoalveolar lavage (BAL) cytokines on day 3 or day 6 post infection. Cytokine concentrations are log-transformed after addition of half of the non-zero minimum (to account for zeros prior to log-transformation), followed by z-score scaling. (A-B; F) Columns represent samples collected from individual mice. Statistical significance for comparisons between genotypes for (F) was assessed using Student’s t test, and p values were adjusted for multiple testing using the FDR approach. *adjusted p < 0.05, **adjusted p < 0.01.
(TIF)

S2 Fig FACS gating strategy and pulmonary immune cell counts. (A) FACS gating strategy for identification of lung cell populations. (B) Percent of activated (CD69^+^) CD4^+^ lung T cells 9 days post infection in WT and *Lcn2*^*-/-*^ mice. (C-E) Neutrophil (C), alveolar macrophage (AM) (D) or B cell (E) counts per lung at indicated timepoints after infection. n = 4 (baseline), 5 (day 9 post infection) and 4–7 (day 16 post infection) per genotype. Bar diagrams show group means +/- SEM, and statistical significance for comparisons between genotypes for (B-D) was assessed using Student’s t test. *p < 0.05.
(TIF)

S3 Fig Antigen presentation capacity of CD103^+^ and CD8a^+^ DCs, overlap of differentially expressed genes in CD103^+^ DCs from infected and uninfected mice and *in vitro* effects of LCN2 deficiency and supplementation. (A-B) CD8^+^ T cell proliferation (assessed by antigen presentation assay, as in Fig 3A) after co-culture with WT or *Lcn2*^*-/-*^ CD103^+^or CD8a^+^ DCs at 1: 100 (DC: T cell) ratio. Replicates of 2 pools per genotype (each consisting of 6–7 mice) are shown. (C) Venn diagram illustrating overlaps in genes up- or downregulated in *Lcn2*^*-/-*^ DCs from infected mediastinal lymph nodes or uninfected lungs. (D) Antigen presentation assay showing numbers of proliferated (proliferation dye^low^) OT-I-specific CD8^+^ T cells after 3 days of co-culture with ovalbumin-pulsed WT or *Lcn2*^*-/-*^ BMDCs. (E) Antigen presentation assay showing numbers of proliferated (proliferation dye^low^) OT-I-specific CD8^+^ T cells after 3 days of co-culture with WT BMDCs, which were pulsed with ovalbumin in presence of recombinant LCN2 at indicated concentrations. Bar diagrams show group means +/- SEM. Statistical significance for (A-B) was assessed using nested ANOVA. Statistical significance for (D-E) was assessed using Student’s t test. n.s. not significant; *p < 0.05.
(TIF)

S4 Fig Effects of LCN2 on microbial richness and diversity. (A-D) Microbiome analysis of bronchoalveolar lavage (BAL), ileal mucosa, ileal lumen, cecum and stool samples derived from 12 weeks old WT and *Lcn2*^-/-^ mice, n = 13–16 per genotype. Shannon diversity (A) and Chao 1 amplicon sequencing variant (ASV) richness (B) for microbial samples from indicated sites. Linear mixed model p values, controlling for cage, are shown. (C) Bacterial phyla with significantly (FDR < 0.1) differential abundance in WT and *Lcn2*^*-/-*^ stool samples. (D) ASVs with significantly (FDR < 0.1) differential abundance between WT and *Lcn2*^*-/-*^ samples from indicated sites along the intestinal tract. SFB: Segmented filamentous bacteria. Boxplots are indicative of median (horizontal line), interquartile range (box) and range (whiskers). Statistical significance for comparisons between genotypes was assessed using linear mixed models on centralized log ratio transformed data, controlling for housing cage.
(TIF)

S5 Fig Antigen presentation after antibiotic treatment and co-housing effects on on the microbiome. (A) Experimental layout for the antigen presentation assay. WT and *Lcn2*^*-/-*^ mice were treated with broad-spectrum antibiotics in drinking water. After four weeks treatment, mice were infected with PR/8-OVA and CD103^+^ DCs were sorted (60 hours post infection) from mediastinal lymph nodes (mLNs), followed by co-culture with purified splenic OT-I T cells. (B) Numbers of proliferated (proliferation dye^low^) CD8^+^ T cells after 96h co-culture (1:100 DC: T cell ratio) with WT or *Lcn2*^*-/-*^ CD103^+^ DCs derived from antibiotics-treated mice. Replicates of 3 pools per genotype (each consisting of 6–7 mice) are shown. (C) Experimental setup for co-housing experiments. WT and *Lcn2*^-/-^ mice were separately- (sep) or co-housed (co) for four weeks prior to stool sample collection. (D) Log_2_ fold change between the phylum *Bacteroidetes* and total bacteria in stool samples, measured by qPCR. Statistical significance for (B) was assessed using nested ANOVA, and for (C) using Student’s t test. n.s. not significant, **p < 0.01.
(TIF)

S1 Table Signaling Pathway Impact Analysis (SPIA) results for CD103^+^ DCs. CD103^+^ DCs were isolated by FACS and processed for RNA sequencing. Related to Fig 1D. This table gives an overview of KEGG pathways associated with differentially expressed genes map, ranked by their global p values. DEG: Differentially expressed gene, tA: net perturbation accumulation, FDR: False discovery rate; FWER: Family-wise error rate.
(XLSX)

S2 Table Signaling Pathway Impact Analysis (SPIA) results for AMs. AMs were isolated by FACS and processed for RNA sequencing. This table gives an overview of KEGG pathways associated with differentially expressed genes map, ranked by their global p values. DEG: Differentially expressed gene, tA: net perturbation accumulation, FDR: False discovery rate; FWER: Family-wise error rate.
(XLSX)

S3 Table 16 rRNA Gene Amplicon Sequencing Variants. Overview of all detected amplicon sequencing variants (ASVs), and their p values for comparisons of ASV abundance between WT and *Lcn2*^*-/-*^ samples. BAL: Bronchoalveolar lavage; NA: not applicable.
(XLSX)

S4 Table Antibodies used for flow cytometry and cell sorting. (XLSX)
