# Supplementary figures and images for: Lipocalin 2 modulates dendritic cell activity and shapes immunity to influenza in a microbiome dependent manner
Source: PLoS Pathog. 2021 Apr 27;17(4):e1009487. doi: 10.1371/journal.ppat.1009487 (PMC8078786; doi:10.1371/journal.ppat.1009487)

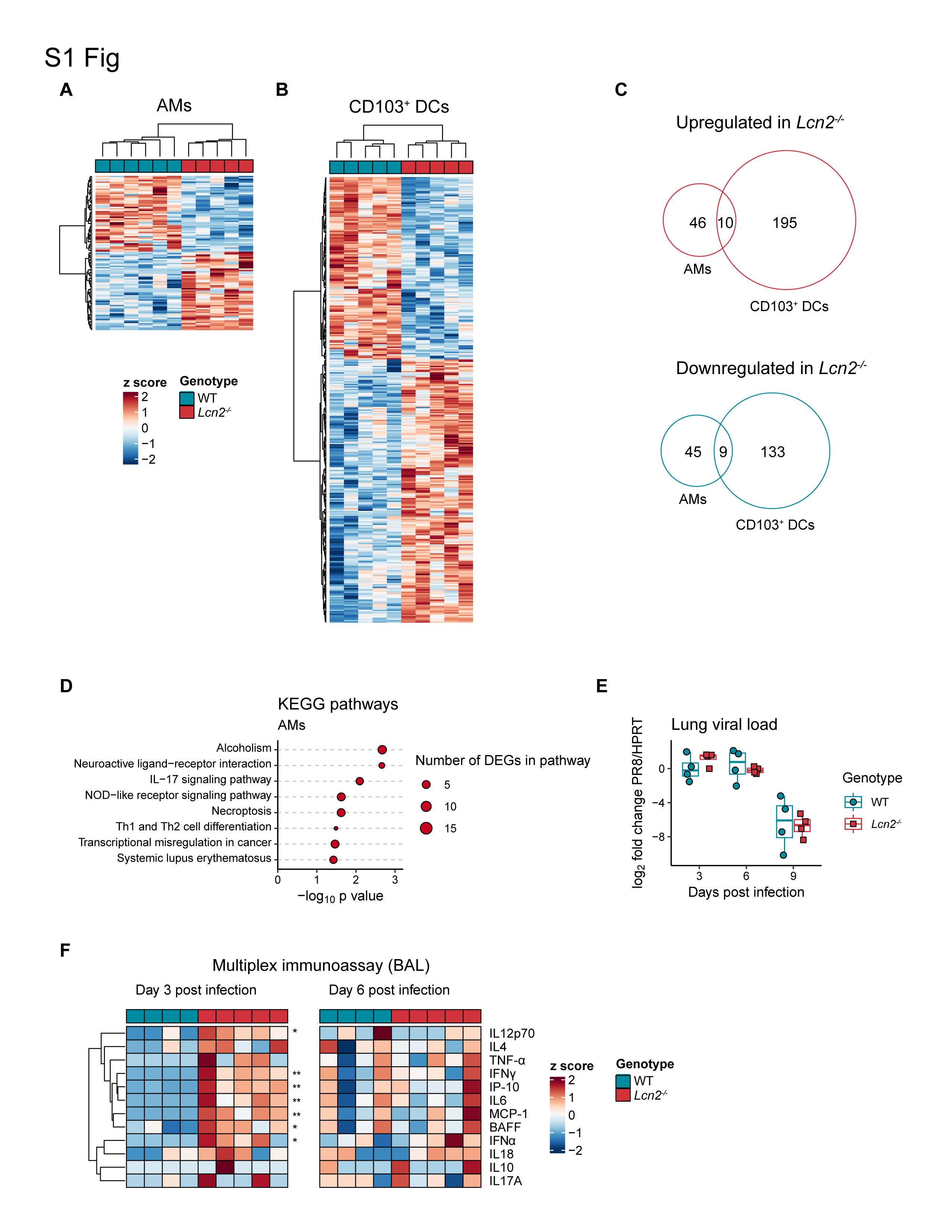

Supplement: S1 Fig — (A-B) Alveolar macrophages (AMs) and CD103+ DCs were isolated from lungs of WT and Lcn2-/- animals by FACS and prepared for RNA sequencing. Heatmaps of differentially expressed genes (DEGs) for AMs (A) and CD103+ DCs (B). Read counts are rlog transformed, followed by z-score scaling. (C) Venn diagrams illustrating overlaps in up- or down-regulated genes according to genotype between AMs and CD103+ DCs. (D) Significantly perturbed KEGG pathways (p value < 0.05) with lowest SPIA (Signaling Pathway Impact Analysis) p values in AMs isolated from mediastinal lymph nodes of PR/8-OVA-infected mice. Circle sizes indicate the number of DEGs associated with the respective pathway. (E) Lung viral load, as measured by qPCR, for WT and Lcn2-/- mice, at indicated timepoints after PR/8 infection. (F) Heatmaps of indicated bronchoalveolar lavage (BAL) cytokines on day 3 or day 6 post infection. Cytokine concentrations are log-transformed after addition of half of the non-zero minimum (to account for zeros prior to log-transformation), followed by z-score scaling. (A-B; F) Columns represent samples collected from individual mice. Statistical significance for comparisons between genotypes for (F) was assessed using Student’s t test, and p values were adjusted for multiple testing using the FDR approach. *adjusted p < 0.05, **adjusted p < 0.01. (TIF) [file ppat.1009487.s001.tif]

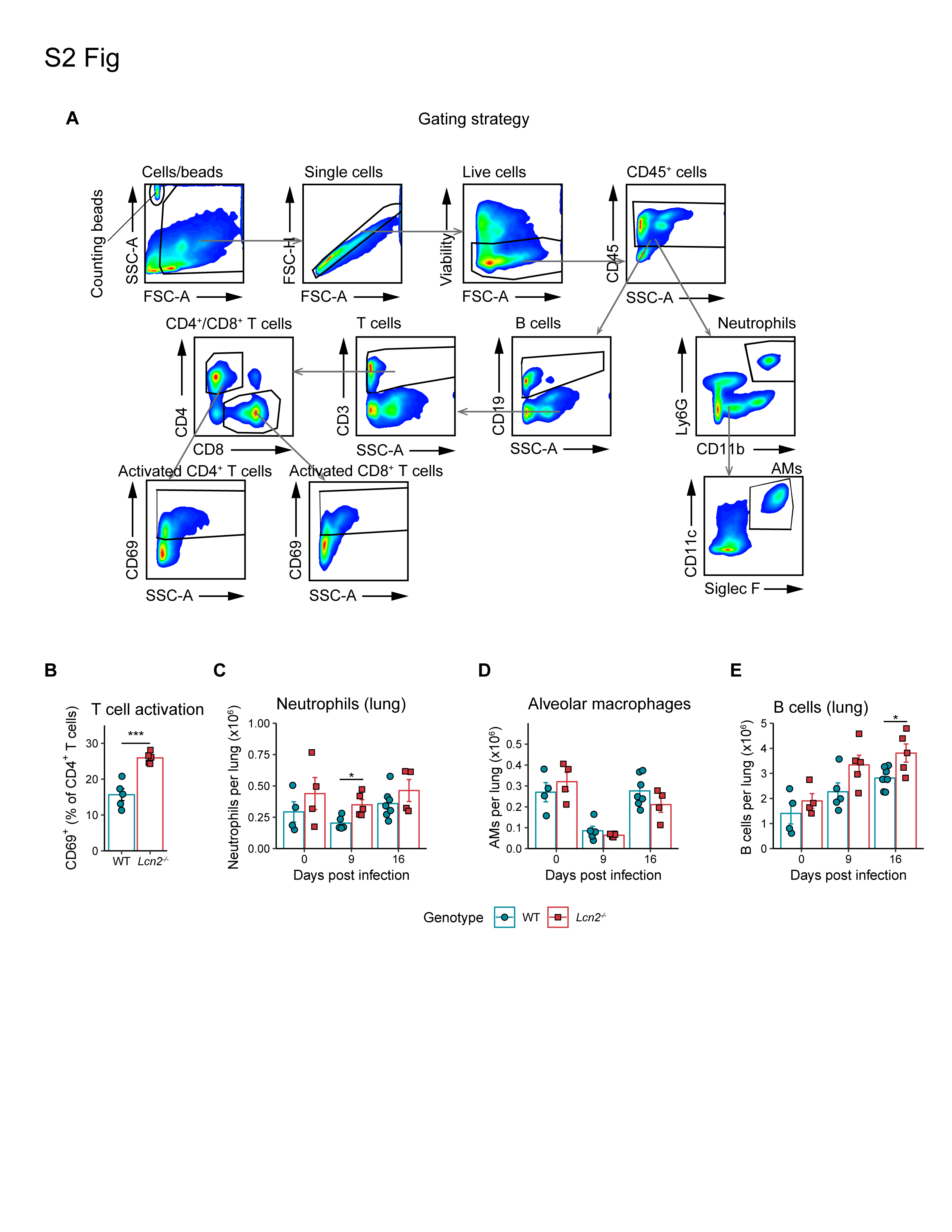

Supplement: S2 Fig — (A) FACS gating strategy for identification of lung cell populations. (B) Percent of activated (CD69+) CD4+ lung T cells 9 days post infection in WT and Lcn2-/- mice. (C-E) Neutrophil (C), alveolar macrophage (AM) (D) or B cell (E) counts per lung at indicated timepoints after infection. n = 4 (baseline), 5 (day 9 post infection) and 4–7 (day 16 post infection) per genotype. Bar diagrams show group means +/- SEM, and statistical significance for comparisons between genotypes for (B-D) was assessed using Student’s t test. *p < 0.05. (TIF) [file ppat.1009487.s002.tif]

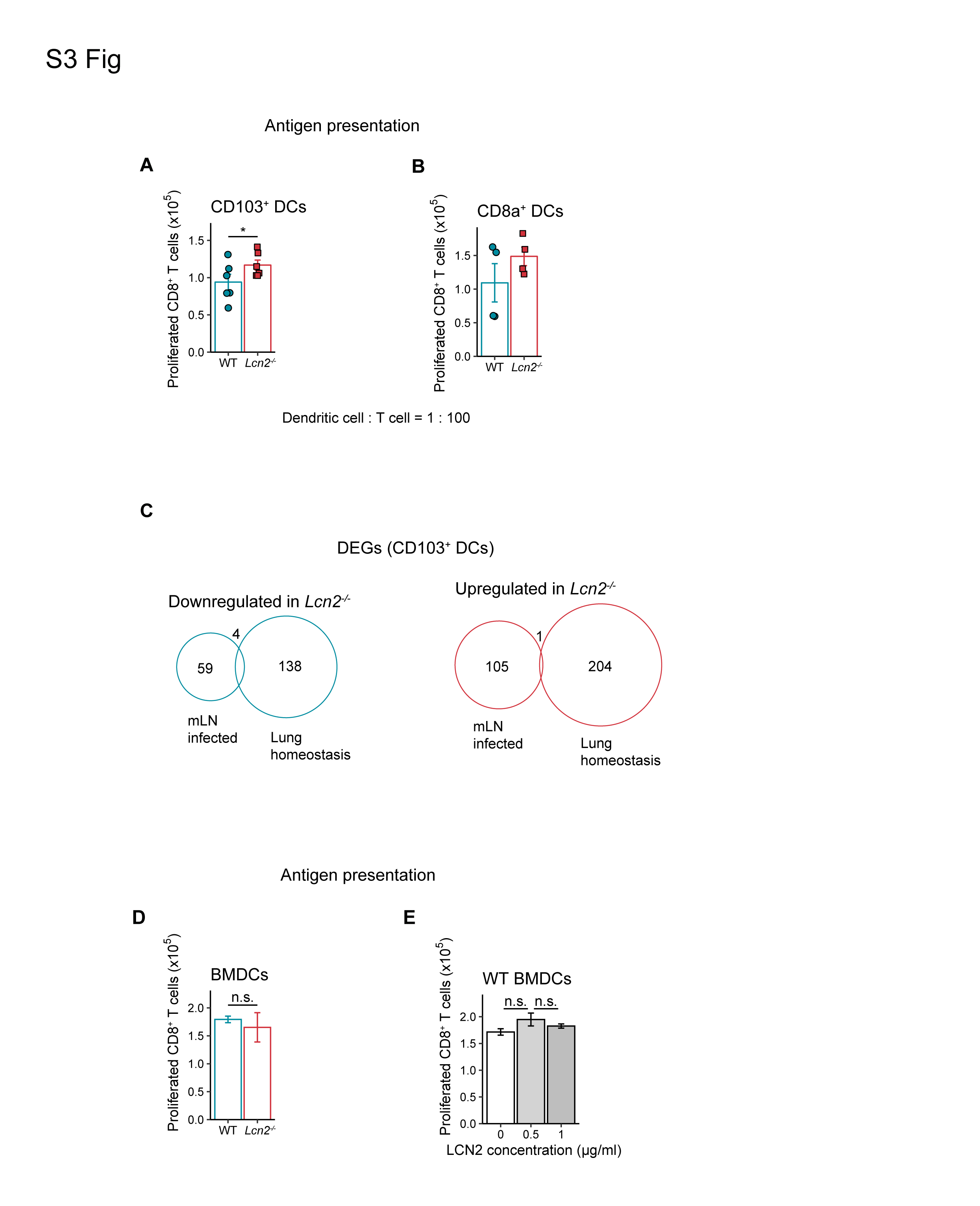

Supplement: S3 Fig — (A-B) CD8+ T cell proliferation (assessed by antigen presentation assay, as in Fig 3A) after co-culture with WT or Lcn2-/- CD103+or CD8a+ DCs at 1: 100 (DC: T cell) ratio. Replicates of 2 pools per genotype (each consisting of 6–7 mice) are shown. (C) Venn diagram illustrating overlaps in genes up- or downregulated in Lcn2-/- DCs from infected mediastinal lymph nodes or uninfected lungs. (D) Antigen presentation assay showing numbers of proliferated (proliferation dyelow) OT-I-specific CD8+ T cells after 3 days of co-culture with ovalbumin-pulsed WT or Lcn2-/- BMDCs. (E) Antigen presentation assay showing numbers of proliferated (proliferation dyelow) OT-I-specific CD8+ T cells after 3 days of co-culture with WT BMDCs, which were pulsed with ovalbumin in presence of recombinant LCN2 at indicated concentrations. Bar diagrams show group means +/- SEM. Statistical significance for (A-B) was assessed using nested ANOVA. Statistical significance for (D-E) was assessed using Student’s t test. n.s. not significant; *p < 0.05. (TIF) [file ppat.1009487.s003.tif]

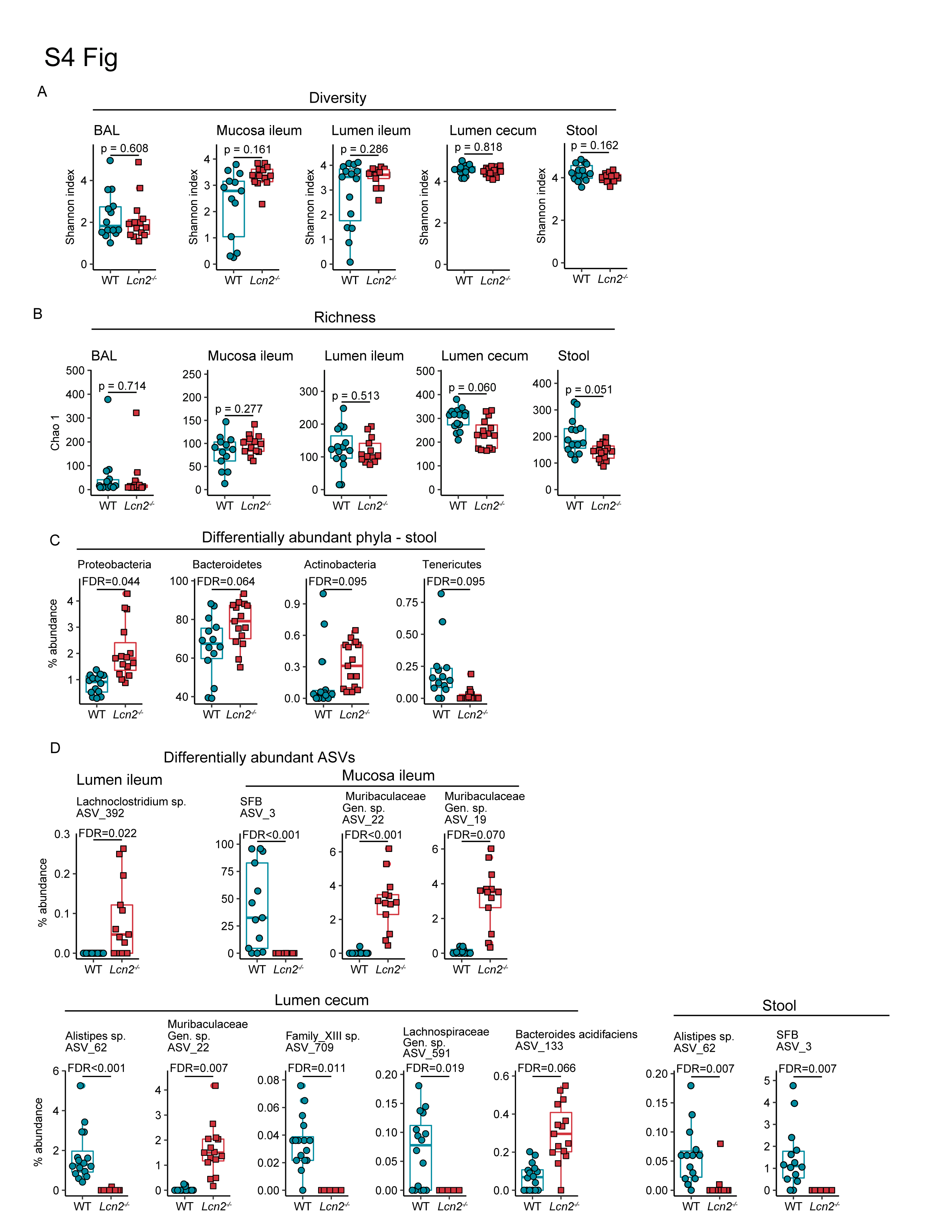

Supplement: S4 Fig — (A-D) Microbiome analysis of bronchoalveolar lavage (BAL), ileal mucosa, ileal lumen, cecum and stool samples derived from 12 weeks old WT and Lcn2-/- mice, n = 13–16 per genotype. Shannon diversity (A) and Chao 1 amplicon sequencing variant (ASV) richness (B) for microbial samples from indicated sites. Linear mixed model p values, controlling for cage, are shown. (C) Bacterial phyla with significantly (FDR < 0.1) differential abundance in WT and Lcn2-/- stool samples. (D) ASVs with significantly (FDR < 0.1) differential abundance between WT and Lcn2-/- samples from indicated sites along the intestinal tract. SFB: Segmented filamentous bacteria. Boxplots are indicative of median (horizontal line), interquartile range (box) and range (whiskers). Statistical significance for comparisons between genotypes was assessed using linear mixed models on centralized log ratio transformed data, controlling for housing cage. (TIF) [file ppat.1009487.s004.tif]

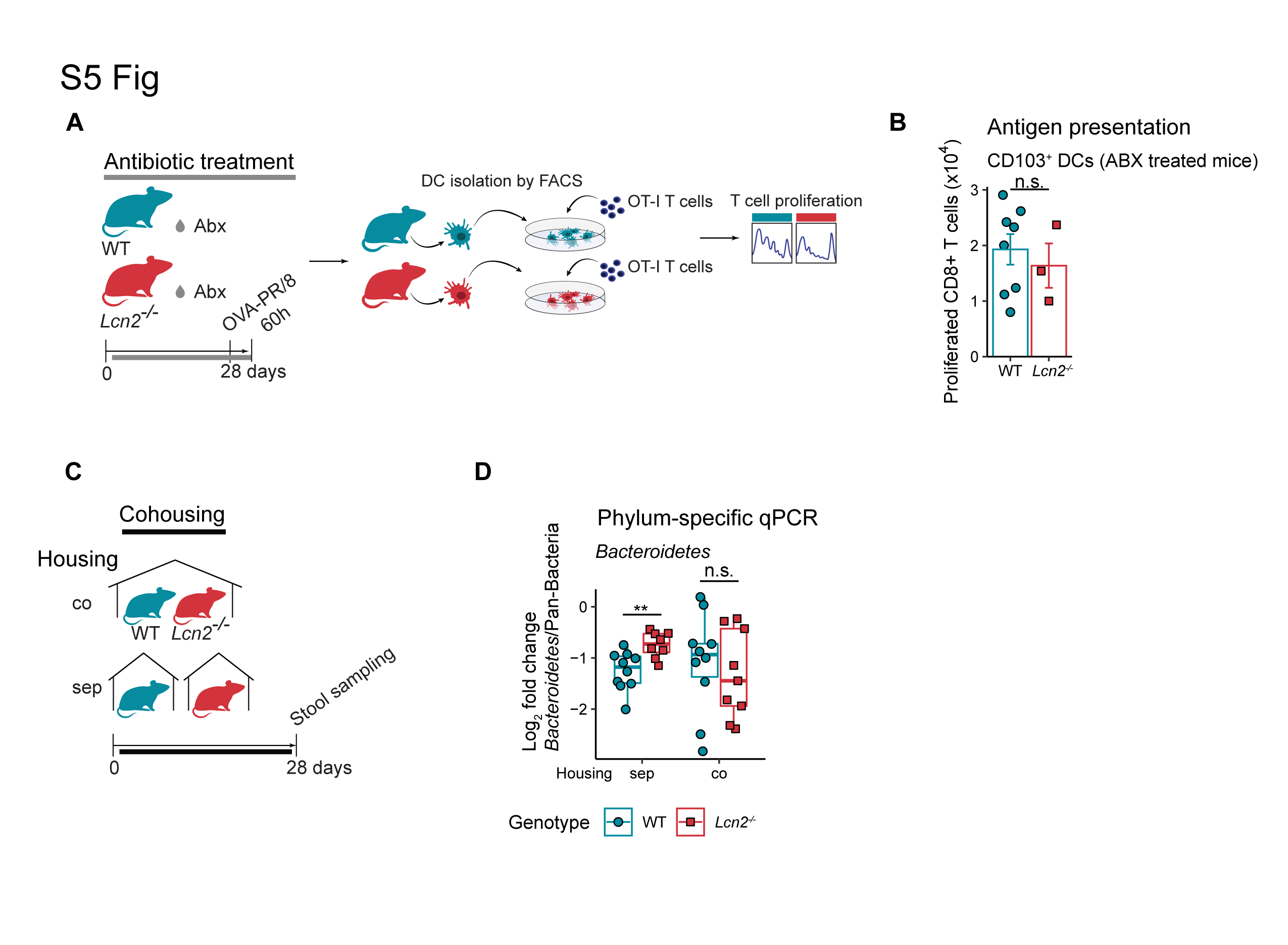

Supplement: S5 Fig — (A) Experimental layout for the antigen presentation assay. WT and Lcn2-/- mice were treated with broad-spectrum antibiotics in drinking water. After four weeks treatment, mice were infected with PR/8-OVA and CD103+ DCs were sorted (60 hours post infection) from mediastinal lymph nodes (mLNs), followed by co-culture with purified splenic OT-I T cells. (B) Numbers of proliferated (proliferation dyelow) CD8+ T cells after 96h co-culture (1:100 DC: T cell ratio) with WT or Lcn2-/- CD103+ DCs derived from antibiotics-treated mice. Replicates of 3 pools per genotype (each consisting of 6–7 mice) are shown. (C) Experimental setup for co-housing experiments. WT and Lcn2-/- mice were separately- (sep) or co-housed (co) for four weeks prior to stool sample collection. (D) Log2 fold change between the phylum Bacteroidetes and total bacteria in stool samples, measured by qPCR. Statistical significance for (B) was assessed using nested ANOVA, and for (C) using Student’s t test. n.s. not significant, **p < 0.01. (TIF) [file ppat.1009487.s005.tif]
